# Shared characteristics of intervention techniques for oral vocabulary and speech comprehensibility in preschool children with co-occurring features of developmental language disorder and speech sound disorder: a systematic review with narrative synthesis

**DOI:** 10.1136/bmjopen-2023-081571

**Published:** 2024-08-28

**Authors:** Lucy Rodgers, Nicola Botting, Sam Harding, Martin Cartwright, Meriem Amer-El-khedoud, Rosalind Herman

**Affiliations:** 1 Department of Language and Communication Science, City University of London, London, UK; 2 Children's Speech and Language Therapy, Sussex Community NHS Foundation Trust, Brighton, UK; 3 Bristol Speech and Language Therapy Research Unit, North Bristol NHS Trust, Westbury on Trym, UK; 4 Department of Health Services Research and Management, City University of London, London, UK; 5 Children's Speech and Language Therapy, Barts Health NHS Trust, London, UK

**Keywords:** speech pathology, developmental neurology & neurodisability, community child health

## Abstract

**Abstract:**

**Objectives:**

To descriptively compare and contrast intervention techniques for preschool children with features of developmental language disorder (outcome: oral vocabulary) and speech sound disorder (outcome: speech comprehensibility) and analyse them in relation to effectiveness and theory.

**Design:**

This is a systematic review with narrative synthesis. The process was supported by an expert steering group consisting of relevant professionals and people with lived experience.

**Data sources:**

Ovid Emcare, MEDLINE Complete, CINAHL, APA PsycINFO, ERIC, and Communication Source from January 2012 were searched. Relevant studies were obtained from an initial published review (up to January 2012).

**Eligibility criteria:**

Interventions for preschool children (80% aged 2:0–5:11 years) with idiopathic speech or language needs; outcomes relating to either oral vocabulary or speech comprehensibility.

**Data extraction and synthesis:**

Searches were conducted on 27 January 2023. Two independent researchers screened at abstract and full-text levels. Data regarding intervention content (eg, techniques) and format/delivery (eg, dosage, location) were extracted. Data were synthesised narratively according to the methods of Campbell *et al*.

**Results:**

24 studies were included: 18 for oral vocabulary and 6 for speech comprehensibility. There were 11 randomised controlled trials, 2 cohort studies and 11 case series. Similarities included a focus on input-related techniques and similar therapy activities. Speech studies were more likely to be professional-led and clinic-led, rather than at home and through a parent. Analysis was restricted by heterogeneity in study design and terminology, as well as gaps within intervention reporting. Information deemed important to the expert steering group was missing.

**Conclusions:**

Similarities and differences between intervention techniques for oral vocabulary and speech comprehensibility have been identified and synthesised. However, analysis of effectiveness was limited due to issues with study design and heterogeneity within studies. This has implications for the progression of the evidence base within the field.

**PROSPERO registration number:**

CRD42022373931.

**STRENGTHS AND LIMITATIONS OF THIS STUDY**

- Relevant electronic databases spanning medicine, education and psychology were searched.
- Key stakeholder engagement has been embedded into all parts of the systematic review process.
- Electronic databases in languages other than English were not searched.
- Not all data could be extracted due to limitations within intervention reporting.
- Analysis of effectiveness was limited by heterogeneity in study design, type of control and outcome measures.

## Introduction

 Within the field of child speech and language disorders, there are often overlapping or co-occurring difficulties that create unique patient experiences. Yet, while there is ample literature on treatment for singly occurring difficulties, there is a notable gap in evidence for treating children with co-occurring disorders. This review focuses on interventions for children who have features of developmental language disorder (DLD) and speech sound disorder (SSD), with the aim of highlighting similarities and differences and informing practice for children where these co-occur.

### Developmental language disorder

Language acquisition is highly variable within the preschool years,^1^ and it has been found that as many as 70% of children who are late to talk (ie, ‘late talkers’) at 18–30 months will catch up spontaneously.^2 3^ Spontaneous recovery is less likely from 3 years, and at this time clinicians may consider that the child has features of a persisting DLD.^4^ An estimated 7.58% of 4-year-old children present with features of a DLD.^5^ DLD is characterised by idiopathic difficulties in using and understanding spoken language,^4^ with a significant impact on a child’s well-being and participation and functioning in life.^6^ Within the preschool years, one feature of DLD is limited vocabulary development.^4^ There is a known association between limited vocabulary development and childhood temper tantrums/mental health, and later language and literacy difficulties into the secondary school years.^7 8^

### Speech sound disorder

SSD is an umbrella term to describe difficulties with the production of speech sounds. An estimated 3.4% of 4-year-old children have idiopathic SSD.^9^ One of the most significant effects of SSD is the impact on a child’s ability to make themselves understood to others in everyday life.^10^ The term for this is speech comprehensibility.^11^ Within speech and language therapy practice, an improvement in speech comprehensibility might also coincide with reduced frustration as the child is more able to effectively express their needs.^10^ It is therefore an outcome of high functional importance for young children with SSD. A related term, speech intelligibility, refers to the acoustic-phonetic decoding of utterances and is very closely related to speech comprehensibility as both are linked to the functional use of speech.^11^ As with limited vocabulary, poor speech comprehensibility/intelligibility within the early years has also been associated with negative longer-term outcomes, including persisting speech difficulties^12^ and poor literacy skills.^13 14^ Although it is typical for very young children not to be fully understood by those around them as their speech develops, by 4 years of age a child would typically be at least 50% intelligible.^15^ Difficulties with comprehensibility/intelligibility may arise from speech disorders that are motor-based (eg, dysarthria) or linguistic-based (ie, phonological). Phonological SSDs are the most frequently presenting SSD subtype^16^ and occur when a child has difficulties with manipulating the different sound contrasts (phonemes) which are needed to form words.^17^ There are different types of phonological SSDs, including consistent phonological disorder (where the child makes consistent sound omissions or substitutions) and inconsistent phonological disorder (where these errors have no consistent pattern).^17^

### Co-occurring DLD and SSD

Overlap between features of DLD and SSD has been evidenced within the preschool years; 36% of 4-year-old children with idiopathic SSD also have oral (ie, expressive-spoken) language features of DLD.^9^ This high rate of co-occurrence is in keeping with historical research in the area,^18^ as well as study data from clinical case loads.^19^ The combined impact of co-occurring features of DLD/SSD is twofold; for example, for children with limited oral vocabulary and speech comprehensibility, not only are they unable to use many words, but the limited words they do have will not be understood to others within their daily lives. It is therefore unsurprising that co-occurring phonological SSD/DLD features in early childhood are associated with negative long-term outcomes relating to literacy^20 21^ and communication,^22 23^ with downstream consequences on quality of life^23 24^ and emotional well-being.^25^ Consequently, access to effective and appropriately targeted intervention for children with this profile is crucial. Such interventions might be grounded in addressing shared difficulties that can present in both disorders.

Research highlights a specific link between DLD and phonological SSDs, as both disorders are underpinned by shared linguistic difficulties.^4^ This overlap is represented in the seminal CATALISE (Criteria and Terminology Applied to Language Impairments: Synthesising the Evidence) DLD consensus paper.^4^ In contrast to phonological SSDs, other SSD subtypes, such as motor-based SSDs like dysarthria, have a less marked overlap with DLD. Although non-phonological SSDs such as articulation disorder and childhood apraxia of speech could also be idiopathic, other non-phonological SSDs often are not. Due to their significant overlap with DLD, which has no known causation, this review will focus on phonological SSDs, which are also idiopathic in nature.

The overlap between language and phonological SSDs is further supported by studies on the speech and language development of young children, where complex and bidirectional relationships between the development of individual sounds (phonology) and words (the lexicon) have been identified.^26 27^ For example, the first words of young children primarily consist of the speech sounds already established within their emerging phonological inventory.^27^ Such findings indicate that this relationship between phonology and the lexicon may have implications for intervention with children with co-occurring features of DLD and a phonological SSD.

### Current interventions for preschool children with co-occurring DLD/SSD

Although this overlap exists between DLD and phonological SSDs, there is currently a paucity of theoretically informed interventions that have been specifically developed for this group,^28^ as well as a recommended dosage for them to be delivered at (eg, number of intervention sessions).

Additionally, published intervention studies primarily target morphosyntactic aspects of expressive language, alongside accuracy of speech sound production.^28 29^ These target areas are not necessarily applicable to all children with co-occurring DLD/SSD features. For younger children with this profile and those whose features of DLD are more severe, building vocabulary is typically targeted in speech and language therapy prior to morphosyntax.^30^ This targeting of vocabulary can also be used as a driver for later sentence development.^31^ Within a child’s everyday life, an improvement in oral vocabulary might be characterised by them using a wider range of words, within different contexts.^32^ Current published interventions aiming to improve oral vocabulary might include direct teaching of specific words,^33^ or the general enrichment of the child’s vocabulary through language facilitation strategies used by the adults around them.^32^ The individual techniques within these published interventions are often used by speech and language therapists within their everyday practice, as evidenced within studies that investigate clinical practice.^34^

‘Child Talk’^34^ was a National Institute of Health Research-funded, large, mixed-methods programme of work in the UK combining quantitative and qualitative data from clinicians with a systematic review that investigated the use of early years’ speech and language therapy interventions. The findings led to the specification of (a) a typology of early years’ speech and language therapy intervention and (b) key intervention ingredients for each typology theme. The Child Talk review identified relevant intervention techniques and activities across their overarching typology, and this included the themes of ‘oral language’ and ‘speech production’. The findings highlighted that for children with co-occurring features of DLD/SSD, clinicians often adapt existing interventions by selecting and combining different techniques for interventions targeting speech *or* language. These language or speech techniques might be grounded in evidence for one or the other, as evidenced in recent systematic reviews for language^32^ or speech.^35^ In the absence of evidence specific to the needs of children with co-occurring SSD/DLD, combining different techniques that are evidence-based for language or speech enables clinicians to use their knowledge and experience to provide the best treatment that they can.^34 36^

Although our knowledge of which techniques work best for children with a co-occurring profile is limited, techniques from language *or* speech interventions might relate to shared underlying theories of potential relevance, as outlined in the following sections.

#### The speech processing model

The speech processing model^37^ posits that a child’s areas of linguistic difficulty might relate to input (ie, listening, processing, understanding) and/or output (ie, motor programming and execution). It also demonstrates the interconnectivity between speech and language within both input (hearing speech) and output (producing speech), with ‘phonological representations’ (speech) and ‘semantic representations’ (language) being unified within the term ‘lexical representations’. Such lexical representations are at the centre of both input and output aspects of speech processing. Within clinical practice, the model might be used to inform therapy approaches; for example, phonological contrast therapy may be selected for children with difficulties with phonological representations.^38^ For the current review, the model is useful in conceptualising where intervention techniques for oral language and speech fall within the input–output chain and for considering the implications of this when delivering a combined speech–language intervention.

#### The lexical restructuring hypothesis

A second linguistic theory for consideration within this review is the lexical restructuring hypothesis. The lexical restructuring hypothesis is a process through which growth in vocabulary has the potential to impact on speech output through the strengthening of phonological representations.^39^ Potential language techniques used within clinical practice that might facilitate both speech and language via the lexical restructuring process are modelling and expanding. Language modelling is characterised by the adult providing the child models of words, without expecting the child to repeat these back.^40^ Language expansions are closely related to this, except that the language modelled includes additional lexical items.^41^ For example, if the child says ‘ball’, the adult might say ‘kicking ball’ back to them. Both techniques are typically linked to growth in expressive language.^41 42^ According to the lexical restructuring hypothesis, language growth due to modelling and expansions has the potential to influence speech sound production through increased accuracy and segmentation of the child’s phonological representations.^39^ This highlights how this theory could be of potential benefit when addressing both speech and language in a child with co-occurring DLD/SSD features.

#### Cognition, neurodevelopment and meaningful interactions

In addition to linguistic theories, theory relating to wider cognition and neurodevelopment is also highly relevant when supporting all children with speech and language needs, including those with co-occurring SSD/DLD features. During early childhood, and indeed throughout life, language learning and use is an integral part of cognitive functioning, which has numerous interlinked components. The heightened influence of these links is evidenced in children with speech and language needs; for example, significant correlations have been evidenced between sustained attention, working memory and language ability in children with DLD.^43^ The multicomponent model of working memory^44^ is an evidence-based and frequently cited model of working memory. Integral to the model is the ‘central executive’, which is primarily responsible for attention control, including enabling the focus of attention and switching attention between tasks.^44^ There are numerous links to language function when adequate attention control is provided through the central executive, most notably via the phonological loop, which first helps to store memory traces in acoustic or phonological form, and then rehearses this memory trace to strengthen it.^44^ In view of this link between attention and language function, it is perhaps unsurprising that evidence suggests that language learning best takes place within interactions that are meaningful for children, whereby they are more likely to sustain their attentional focus.^45^ This link between attention and language via meaningful interactions has also been reflected in neuroimaging studies, where Broca’s area has been shown to become activated in response to a child being exposed to meaningful back and forth interactions, rather than in response to passively ‘hearing’ words.^46^ Therefore, within a combined intervention for children with co-occurring features of SSD/DLD, the way in which techniques are delivered (ie, within an engaging activity, which the child can put their attentional focus on) may be just as important as the techniques themselves.

In summary, these three theoretical considerations highlight a valuable opportunity for interventions specific to young children with co-occurring features of DLD/SSD to be developed, using techniques and aspects of their delivery that can be supported by the relevant theory. Due to the current paucity of evidence, the associated negative impact of this co-occurring profile on long-term outcomes and the high level of presentation on clinical case loads, there is an urgent need for such intervention development to take place. The first stage in this process is to conduct a systematic review to identify potential techniques of relevance.

### Study overview and outcomes

Both DLD and SSD are heterogeneous disorders^4 17^ and therefore have a range of associated outcomes. This review will focus exclusively on the outcomes of oral vocabulary (DLD outcome) and speech comprehensibility (SSD outcome) due to the aforementioned impact of such difficulties on the everyday lives of young children. This decision is elaborated on in the Patient and public involvement section of this paper.

The dose form framework^47^ has been used to guide the intended shared characteristics for comparison. Unlike other commonly used intervention frameworks such as the Template for Intervention Description and Replication (TIDieR),^48^ the dose form framework is specific to paediatric speech and language therapy. It therefore contains additional information of relevance to the paediatric speech and language therapy context, such as the location of an activity within a child-centred, clinician-directed continuum.

Shared characteristics for DLD/phonological SSD intervention techniques may include similarities in the following:

1. Who delivers the technique; for example, the parent, clinician or both.
2. Where the technique is delivered; for example, at home, nursery, clinic or a combination of these.
3. The nature of *technique delivery*; for example, whether the activity is presented in an adult-led structured game, play, everyday routines or a combination of these.

Underpinning theory may relate to the following:

1. The lexical restructuring hypothesis.^39^
2. Psycholinguistic models of speech and language development, such as the speech processing model.^37^
3. Cognition (multicomponent model of working memory) and neurodevelopment, specifically the role of attention and meaningful interactions within language learning.^44 46^

Synthesis of dosage will incorporate dose frequency (eg, how many intervention sessions are there, how many times a technique is used within an activity or session) and total duration (eg, length of individual intervention sessions as well as the amount of time between the onset and completion of the intervention).^49^

Intervention techniques will be linked by the research team to relevant theories to address the research questions.

### Research questions

The objective of this review is to compare and synthesise evidence from SSD and DLD interventions in order to inform future intervention development. Specifically, the following are the research questions:

1. What are the shared core characteristics of intervention techniques in preschool interventions targeting speech comprehensibility and/or oral vocabulary?
2. How do these shared core characteristics relate to underlying theory?
3. What evidence is there on the effect of interventions that incorporate these core characteristics of intervention techniques?

This review builds on the previous systematic review for Child Talk.^34^ One of the authors of the current review (SH) led the systematic review programme of work within Child Talk. The current review not only updates that search, but also focuses in more depth on oral vocabulary and speech comprehensibility intervention techniques by relating them to underlying theory. This enables a specific contribution to the literature that is more relevant to the outcomes and children of interest within the current review.

## Methodology

The systematic review was registered on the International Prospective Register of Systematic Reviews (PROSPERO) on 16 December 22 (registration number CRD42022373931). The registered protocol for the current review was peer-reviewed and is available open access.^50^ There were two subsequent amendments to the methodology, which are outlined in the ‘Risk of bias assessment’ and ‘Patient and public involvement’ sections. All other key information, including population, comparator and outcomes eligibility criteria, remains as presented within the published protocol.^50^

### Eligibility criteria

The eligibility criteria are in line with the criteria from the original Child Talk systematic review^34^ and are elaborated on in the following sections. Some amendments to the search strategy (online supplemental material 1) were made according to the objectives of the current review, that is, focusing specifically on the ‘expressive language’ and ‘speech’ themes generated from Roulstone’s^34^ initial typology of preschool speech and language therapy interventions, as these themes encompass the two outcomes for which intervention techniques were sought.

#### Study designs

Included studies were required to report on an empirical evaluation of the effectiveness of an intervention. To ensure identification of all relevant literature, a range of study designs were included, for example, randomised controlled trials (RCTs), experimental and quasi-experimental studies, within-subject designs (eg, pre–post studies) and case studies (which may include multiple baseline or other systematic manipulation of the intervention). Studies that reported on a single timepoint (eg, cross-sectional studies) were excluded. Studies focusing on efficacy, including lab-based training, were not excluded where all other inclusion criteria were met. This is because information on the efficacy of speech/language learning techniques could be gleaned from these studies.

#### Population

To capture the age group most typically seen within preschool clinical services, studies needed to have at least 80% of sample children aged between 2:0 and 5:11 years. The children within the included studies must have presented with phonological speech production difficulties and/or difficulties relating to oral vocabulary, with all subtypes of phonological SSD included (ie, consistent and inconsistent phonological disorder, phonological delay). These difficulties could have been identified by standardised assessments such as the Preschool Language Scale,^51^ parental and/or professional observation reports such as the Intelligibility in Context Scale^52^ and/or probes. As observed in the literature, common probes within speech and language therapy interventions may include a selection of words containing the child’s targeted speech sound/s or vocabulary.^53 54^ Probes may also have been used to assess progress through the repeated measurement of the dependent variable before, during and after the intervention. In keeping with the diagnostic description within the CATALISE article,^4^ included papers had to state that the participants’ needs had no obvious cause, that is, they excluded children with neurodevelopmental differences that have a known association with speech and/or language development, such as autism or cerebral palsy. Due to the challenges in diagnosing DLD in very young children,^4^ and to maximise the identification of potentially relevant intervention techniques, studies were included where a child did not have a formal diagnosis of DLD but was described as a late talker.

#### Intervention

Included studies could report on interventions delivered in any setting (eg, home-based, clinic) or format (eg, face-to-face, online). The deliverer could be a speech and language therapist, speech and language therapy assistant or equivalent professional (including education staff), and the intervention could involve professionals training others (eg, parents) to deliver some or all of the intervention.

#### Comparator

Comparators for included studies could be a control group who did not receive an intervention (including multiple baseline and within-subject designs) or an alternative experimental group (ie, intervention comparison).

#### Outcomes

Included papers had to measure the effectiveness of the intervention on (a) oral vocabulary and/or (b) speech comprehensibility. These outcomes had to be evaluated via standardised assessments, probes and/or observational ratings or scales.

If composite speech and language assessments were used, studies had to report on the separate subtest results for oral vocabulary and/or speech comprehensibility to be included.

Studies with only syntactic measures of language change were excluded; this included mean length of utterance in morphemes. However, they were still included if a proximal measure of vocabulary change was used alongside syntactic measures, such as the number of different words. It is possible that oral vocabulary studies will incorporate outcomes relating to both ‘static’ and ‘fluid’ vocabulary. We defined static vocabulary as specific words that are elicited in response to a set stimulus (eg, the child being asked to label what they see in a picture). In contrast, we defined fluid vocabulary as more flexible, where the vocabulary is spontaneously uttered by the child (eg, through their comments in child-led play). Although static and fluid vocabularies are slightly different constructs, for the purpose of this review we included studies that measure outcomes in either, to capture as much potentially relevant data as possible.

Speech comprehensibility is the SSD outcome in focus. As previously mentioned, comprehensibility and intelligibility are overlapping but differing constructs, with a shared focus on functional human communication.^11^ Therefore, we also included studies with an outcome of improved speech intelligibility as a proxy for comprehensibility. This was deemed more suitable than using measures such as percentage of consonants correct as a proxy for comprehensibility, where the focus was more on speech accuracy. Due to the recent consensus in terminology, measures for comprehensibility could include measures with ‘intelligibility’ within their title, such as the ‘intelligibility in context’ scale, which is becoming increasingly used in SSD intervention research.^52^

### Information sources, search strategy and selection

The Child Talk review search strategy^34^ was updated for the current review, accounting for advances in terminology, for example, consensus on the term ‘developmental language disorder’.^4^ Child Talk also encompassed a broader range of speech and language outcomes; therefore, the search terms for the current review were adjusted to focus on our two specific outcomes of interest: oral vocabulary and speech comprehensibility. The updated search strategy was initially reviewed by two independent postdoctoral researchers in the field and adjusted as needed, for example, adding in the term ‘specific language impairment’, which may be relevant to older papers in the search. The final search strategies for each database can be found in online supplemental material 1.

The searches were conducted on 27 January 2023 using the databases APA PsycINFO, Communication Source, CINAHL, ERIC, MEDLINE Complete and Ovid Emcare. Identified records were uploaded to RefWorks and duplicates were removed. ERIC records dated pre-January 2012 were also removed in RefWorks as this function was not available on the ERIC database. The remaining studies were then uploaded to Covidence software; Covidence is a web-based collaboration software platform that streamlines the production of systematic and other literature reviews.^55^ Potentially relevant papers from the original Child Talk review were also identified from the original Child Talk extraction form (Excel spreadsheet), filtering for the two outcomes of interest for the current review. Potentially relevant papers identified from the original Child Talk review, supplementary screening of reference lists and screening of reference lists from recent related reviews^56 57^ were sent straight to full-text screening. Due to resource constraints, articles written in languages other than English were excluded. However, articles written in English where the participants spoke languages other than English were included. Additionally, grey literature searching was confined to the inclusion of theses/dissertations, via the above-stated databases.

The screening and data extraction were carried out as follows:

Initially the first author (LR) trialled an exclusion guidance document on 30 papers. These 30 papers were then screened by a second independent reviewer (MA-E-k). Discrepancies in the selection and use of the document were discussed, with amendments made following this.

1. Abstract and full-text screening of all papers was then carried out by two independent reviewers (LR and SH). Prior to consensus meetings, Cohen’s kappa was moderate (0.49; 91% agreement) at abstract screening and fair (0.33; 73% agreement) at full-text screening. At full-text screening, 84% of conflicts arose due to a ‘maybe’ vote being paired with a ‘yes’ or ‘no’ vote. Full agreement was reached within consensus meetings.
2. Retained studies then underwent quality appraisal by two reviewers (LR and SH). The reviewers had regular consensus meetings, after independently assessing up to four papers at a time; agreement was reached on all items.
3. The two reviewers (LR and SH) independently extracted data from a random selection of 25% of studies. Agreement was reached on all extraction points. One reviewer (LR) then completed the remainder of the extraction.

#### Data items

In line with the registered and published protocol for the present review,^50^ data items for extraction included overarching study details, intervention details (eg, techniques, location, deliverer) and outcome information. Full data items are specified in online supplemental material 2.

#### Risk of bias assessment

To encompass the range of study designs included within this review, the PEDro-P (Physiotherapy Evidence Database-PsychBITE) was used.^58^ For single-case experimental designs, the RoBiNT (Risk of Bias in N-of-1 Trials) scale was used.^59^ In a deviation from the initial protocol, the threshold for inclusion within the analysis of effectiveness following quality appraisal was amended to 50% for internal validity on both RoBiNT and PEDro-P. This calculation excluded items relating to blinding of participants and interventionists, which cannot be achieved due to the nature of speech and language therapy interventions. Descriptive information is provided for all studies within the results; however, only studies meeting the 50% threshold for internal validity were planned to be used in synthesis relating to effectiveness. The researchers acknowledge that risk of bias has the potential to vary considerably among the studies. This includes studies reaching the threshold of 50% or above for analysis of effectiveness. To aid transparency with this process, the risk of bias ratings for each individual study are available through supplementary materials.

Reporting bias was also assessed by first identifying if protocols existed for each study. When not, the outcomes and the results were compared for selective reporting bias. The two reviewers (LR, SH) reached 100% agreement on this with a random selection of 25% of papers; LR completed the remainder.

#### Synthesis methods

In line with the registered and published protocol,^50^ narrative synthesis was planned due to the anticipated heterogeneity in study design and effect measures.^60^ Synthesis without meta-analysis in systematic review guidelines^61^ was used to guide this. It was intended that the narrative synthesis would have sections regarding similarities and differences between intervention techniques, patterns in technique dosage and delivery across the interventions, and how they related to underlying theory. As stated in the registered protocol for the present review, challenges identified regarding the gaps and quality of the knowledge base were also to be presented if relevant.

### Patient and public involvement statement

Outcomes were prioritised by clinicians and parents of preschool children with DLD/SSD within prestudy patient and public involvement (PPI) work. This consisted of brainstorming sessions within PPI parent advisory group meetings and feedback from 86 clinicians from across 30 different UK National Health Service trusts via Clinical Excellence Network meetings.^62 63^ The outcomes of increasing (a) oral vocabulary (*DLD outcome*) and (b) speech comprehensibility (*SSD outcome*) were identified as priorities. This provides further rationale for focusing on techniques that directly target oral vocabulary and speech comprehensibility.

This review was overseen by a newly formed steering group consisting of professionals (speech and language therapists, a clinical equality diversity and inclusion expert, a specialist early years teacher, a bilingual/multilingual support worker) and people with lived experience (a parent of a child with DLD/SSD, an adult with DLD). The Cochrane ‘Involving People’ resource was used to guide their involvement.^64^ The overarching aim of steering group involvement was to enhance social validity through integrating a diverse range of ‘real life’ perspectives and experiences into the conduct and analysis of this review. Input consisted of individual meetings with the researcher and two whole group meetings. Group members highlighted areas of importance to relevant professionals and people with lived experience, such as the child’s view of the intervention (eg, Was it fun?), their attention and engagement levels, and how bilingual/multilingual children were assessed. This led to a second deviation from the protocol, where additional items were added into the data extraction process (online supplemental material 2). Preliminary results of the review were shared with the group and used as a basis for discussion and brainstorming and have been incorporated into the Results and Discussion sections of this paper. This largely centred on the lack of reporting of information they deemed important and the impact of this on clinicians being able to implement the interventions described. Further information on steering group involvement has been reported using the Guidance for Reporting involvement of Patients and the Public-2 Short Form^65^ (online supplemental material 3).

## Results

### Overview of study characteristics

**Figure 1:**
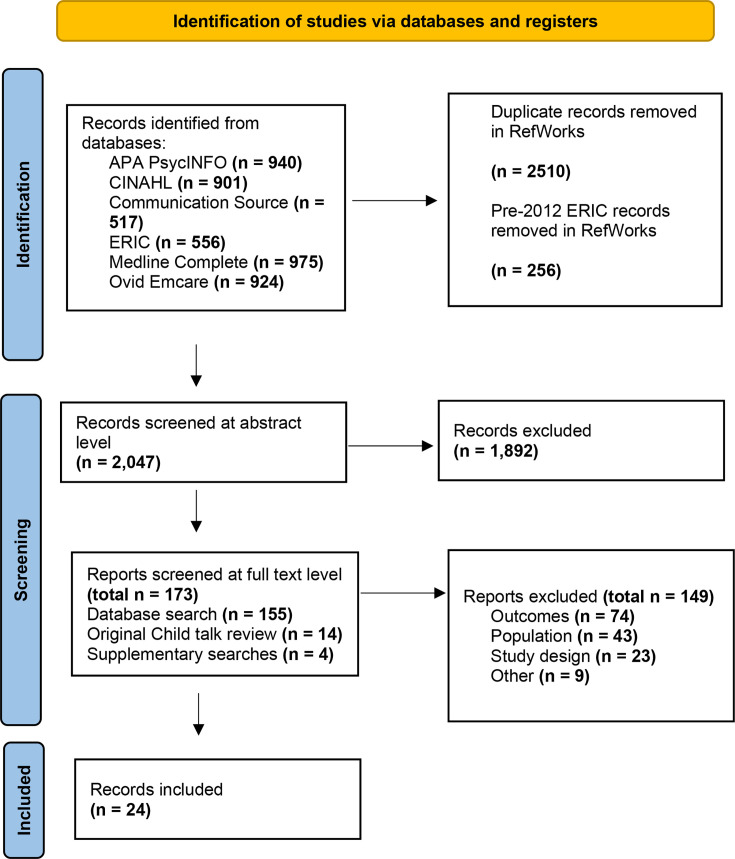
PRISMA flow diagram. PRISMA, Preferred Reporting Items for Systematic Reviews and Meta-Analyses.

The number of records identified and included/excluded at different points in the screening process is displayed in the Preferred Reporting Items for Systematic Reviews and Meta-Analyses flow diagram (figure 1). The screening process resulted in 24 included studies: 11 RCTs,^66^,^76^ 2 cohort studies,^77 78^ 2 case series (alternating treatments),^79 80^ 7 case series (multiple baseline),^5481^,^86^ 1 case series (descriptive cases taken from a larger trial)^87^ and 1 case study (multiple baseline).^88^ The 24 papers reported on 28 interventions in total, with 4 studies comparing 2 different interventions with a control.^66 67 70 73^ Six studies were based on named intervention programmes: Hanen Target Word^69^ and enhanced milieu teaching.^7172 82^,^84^ Many techniques stated within these intervention programmes were not exclusive to these interventions alone.

Eighteen studies had an outcome related to oral vocabulary^66^,^7377^ and six had an outcome relating to speech comprehensibility.^74^,^7686 88^ No studies included outcomes for both. Three of the RCTs randomised participants into alternative treatment groups, rather than a control group.^66 67 70^ Due to this heterogeneity in study design and controls, informal methods have been used to investigate the heterogeneity in reported effects, including the ordering of studies by study design in online supplemental material 4, and the grouping of reported effects according to study design and intervention type (speech or language) within the narrative synthesis.

Tables providing an overview of the included studies can be found in online supplemental material 4.

### Bias

Due to the nature of speech and language therapy interventions, no studies met the criteria pertaining to the blinding of participants or interventionist. These items are therefore excluded when giving the following percentages for internal validity. Overall percentages for the internal validity ratings ranged from 56% to 100% for studies assessed using the PEDro-P. Internal validity within the case series designs was notably low; 6 out of the 10 studies did not meet the 50% threshold for synthesis of effectiveness, even when the questions relating to the blinding of participants/interventionists were discounted. Items 1 and 2 (design with control and randomisation) were only partially present in one of the seven studies.^54^ Full details of internal validity ratings can be found in online supplemental material 5.

Regarding meta-bias, 2 of the 24 papers referred to a published protocol,^72 86^ both of which were registered prior to recruitment. For the remaining 22 papers, the outcomes and measures stated in the methodology and subsequent reporting within the results sections did not differ.

### Synthesis of search results

#### Question 1: what are the shared core characteristics of intervention techniques in preschool interventions targeting speech comprehensibility and/or oral vocabulary?

##### Deliverer and setting

**Figure 2:**
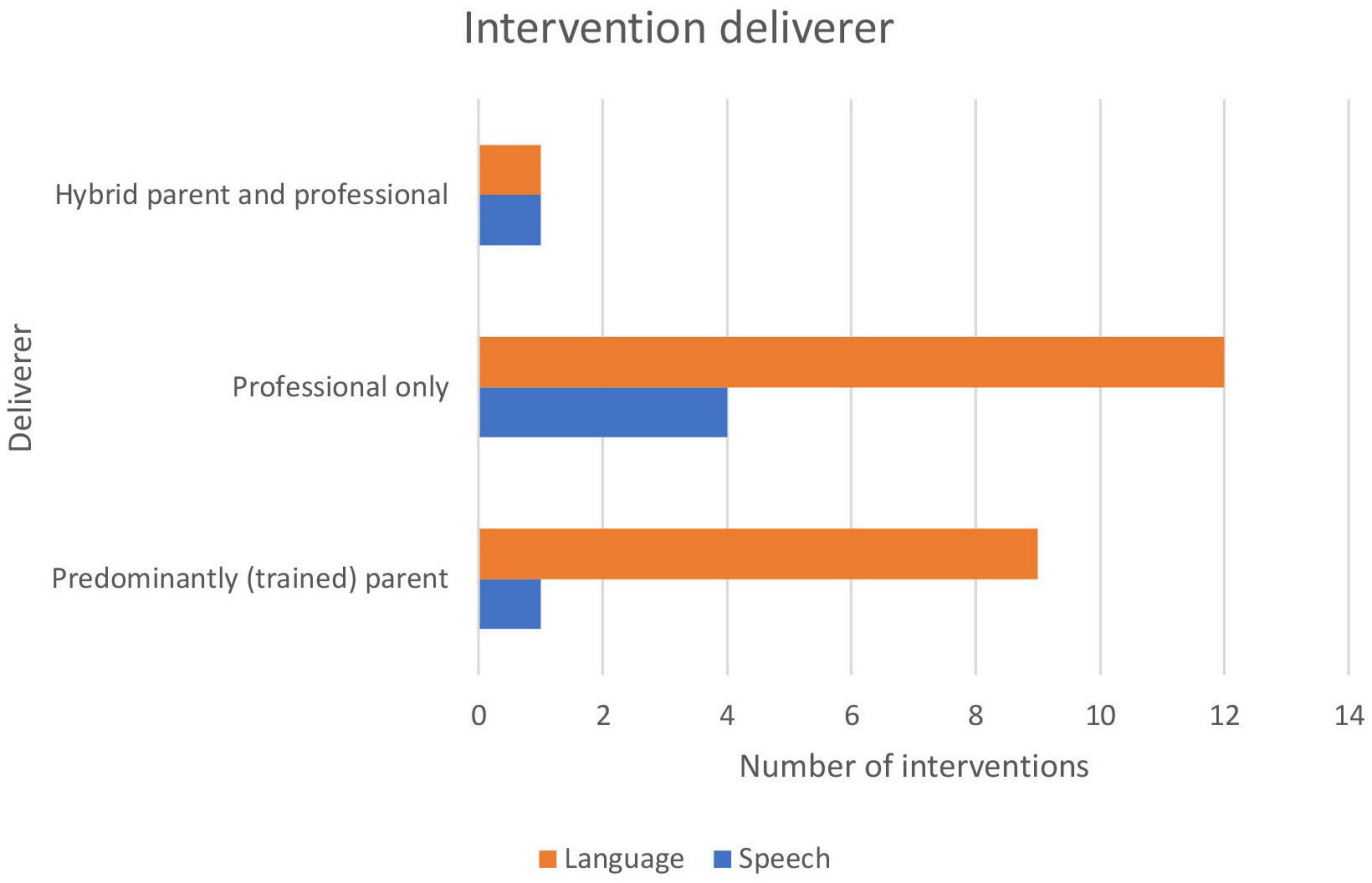
Comparison of intervention deliverer across the speech and language interventions.

For both speech and language interventions, delivery by a professional, and second with the parents, was the most common. Four speech interventions^75 76 86 88^ and 12 language interventions^6667 70 73 78^,^81 85 87^ were delivered solely by a professional. Relative to speech interventions, language interventions often involved delivery via trained parents, with two speech interventions^54 74^ and ten language interventions^67^,^6971^ involving some delivery via trained parents. A comparison of deliverer across the speech and language interventions can be found in figure 2. This delivery via trained parents addresses known associations between parental language responsiveness/input and later language development.^89^ Although delivery of speech intervention by parents can enhance dosage, these findings reflect the known complexity of speech interventions and barriers to skilling up non-professionals to deliver them.^90 91^

**Figure 3:**
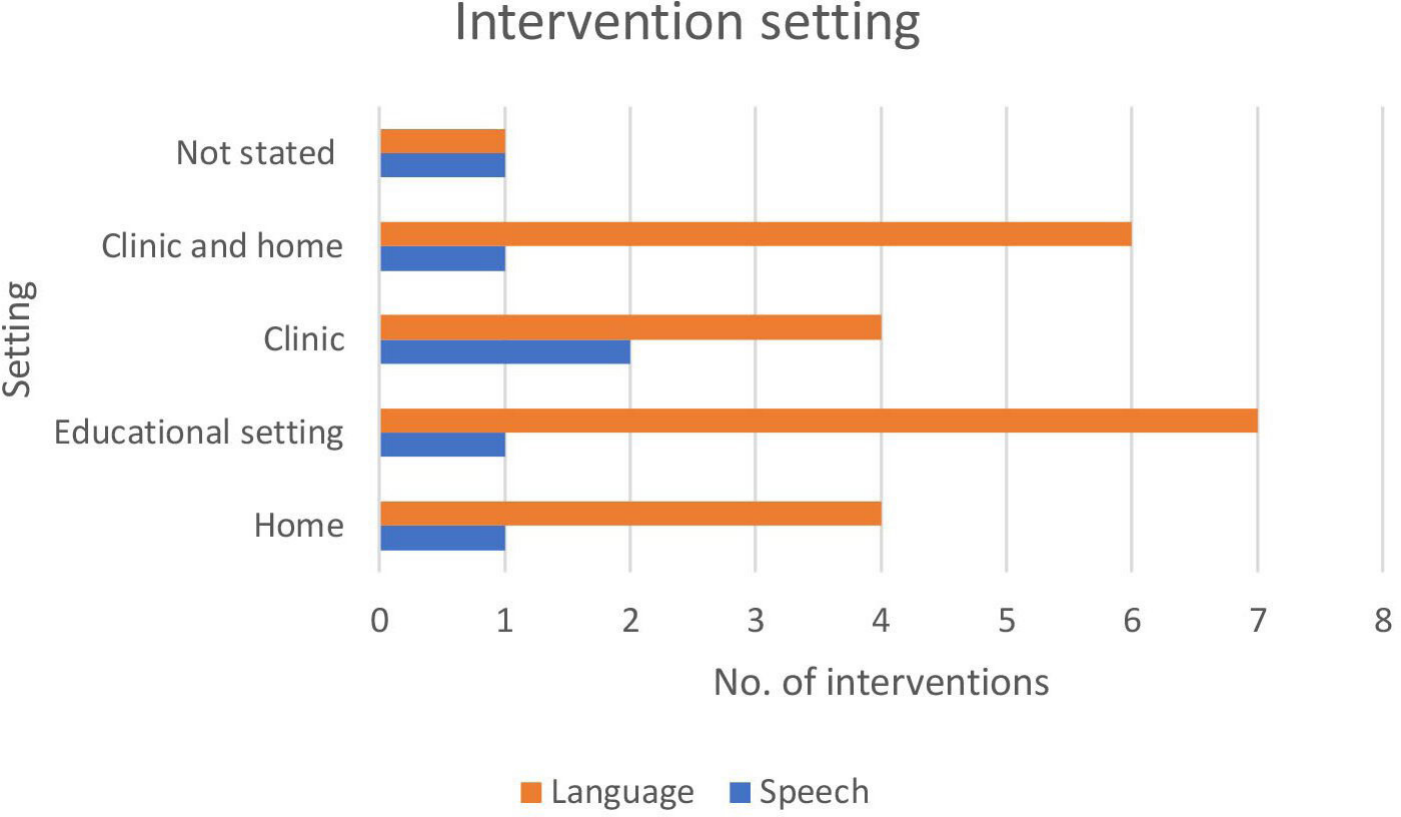
Comparison of intervention location across the speech and language interventions.

One speech intervention^74^ and four language interventions^68 79 82 83^ took place at home. One speech intervention^75^ and seven language interventions^66 70 78 81 85 87^ took place in an educational setting. Two speech interventions^76 88^ and four language interventions^66 67 73^ took place in a clinic. One speech intervention^86^ and one language intervention^80^ study did not state the location of the intervention. A comparison of intervention location across the speech and language studies can be found in figure 3. The interventions taking place at ‘clinic and home’^54 67 69 71 72 77 84^ were typically characterised by the professional training the parent in clinic to continue with intervention techniques at home.

One model of parent training was mentioned in a speech study: the five principles of ‘joint planning-observation-action-reflection-feedback’.^54^ Two specific models of training parents were referred to in the language studies: ‘teach-model-coach-review’^71 72 83 84^ and ‘prepare-present-practice-personalise’.^69^ Although they use different terms, these three methods of training parents have a shared emphasis on the parent practising what has been learnt, opportunities to reflect on what has been learnt/applied and an explicit connection between intervention techniques taught and how to use them at home.

##### Techniques and activities

Modelling was the most frequently mentioned technique within the language interventions, with 15 of the 22 language interventions naming this.^66^,^6870 71 77^ One example of how this technique was operationalised was through the naming of items according to the child’s attentional focus within an activity.^77^ Expansion was the next most common technique, included in 9 out of the 22 language interventions.^6869 71 72 77 78 82^,^84^ One example of how this technique was operationalised was by expanding on the child’s message during free play and child-led activities and daily routines by adding one or two words.^69^

For the speech interventions, two studies did not specify particular techniques.^75 88^ Of the remaining four interventions, the two most frequently referred to techniques included recasting^74 86^ and drill play (ie, providing multiple opportunities for the child to hear and produce the sound).^54 86^ A list of all the techniques extracted is provided in online supplemental material 6. There was variability observed in the labelling of intervention techniques within both speech and language interventions. For example, the technique of waiting was also described as ‘wait and listen’,^69^ ‘wait time’^78^ and ‘expectant pause’.^79^

There were similarities regarding the activities in which the techniques were used for both speech and language interventions, as highlighted in table 1.

**Table 1:** Most common activities within speech and language interventions

Activity	Speech interventions (percentage)	Language interventions (percentage)
Daily routines	1 (17)^74^	8 (36)^67^,^6971 72 83 84^
Play	4 (67)^54 74 76 86^	14 (64)^6668 69 71^,^73 79 81^
Books	2 (33)^54 74^	10 (45)^6670 71 77 78 83^,^85^

There was diversity in how play was characterised within the interventions, with 11 interventions describing fully child-led/naturalistic play^6668 69 71 72 76 79 82^,^85^ and 6 describing play with varying levels of adult structuring.^54 73 74 81 86^ Three of the interventions with structured play were speech interventions, which corresponds to the finding that speech interventions were typically more professional-led and in clinic. For the speech interventions included within this review, this adult-led, more structured play enabled the interventionists to provide the optimum environment for exposure to specific sounds.^54 74 86^

### Question 2: how do these shared core characteristics relate to underlying theory?

#### The speech processing model

The most frequently used intervention techniques for both speech and language related to the input aspects of the speech processing model. Comparatively, output techniques were more present within the speech interventions overall. Table 2 lists the top four techniques; see online supplemental material 6 for the full list.^37^

**Table 2:** Techniques and the speech processing model

Top four techniques	Speech or language	Focus
Modelling	Language	Input
Expansions	Language	Input
Recasting	Speech	Input
Drill play	Speech	Hybrid: input and output

The input techniques (modelling, expansions and recasting) present numerous demands on speech processing; peripheral auditory processing is required prior to later speech/non-speech discrimination and phonological recognition. By contrast, drill play, a mixed input/output technique, taps into all parts of the speech processing model from peripheral auditory processing through to motor execution. As the model proposes, when learning both sounds and words, lexical representations can be strengthened through focusing on both input and output. However, some additional considerations need to be given regarding speech. For example, when using techniques designed to elicit output, potential reinforcement of incorrect motor patterns may arise from the child repeatedly rehearsing an incorrect form of the word. Details regarding the child’s stimulability of their speech targets were limited across the studies, although two studies^54 86^ did refer to this when selecting speech targets.

#### Measuring vocabulary and the lexical restructuring hypothesis

Outcome measures for oral vocabulary were heterogeneous and included (a) assessment of specific targeted words, through probes or from a language sample^70 73 79 84 85 87^; (b) formal standardised assessment^66 67 69^; (c) assessment of lexical diversity within a language sample (number of different words and mean length of utterance in words)^6871 73 77 78 80^,^82^; and (d) parent report of language use via standardised measures^68 72^ or subjective word lists.^67^ Four studies included a combination of two or more of these four types of measure.^67 68 72 73^ This diversity in vocabulary measures has implications for theory development. When using the lexical restructuring hypothesis to investigate concurrent improvements in speech and language, the evidencing of wider lexical diversity, rather than specifically targeted words, has the potential to be more informative by more flexibly capturing broader, system-wide change.^92^ Although the included studies did not aim to explore the lexical restructuring hypothesis, this is an important consideration that can be taken forward within interventions yet to be developed.^39^

#### The multicomponent model of working memory: cognition, neurodevelopment and meaningful interactions

Sixteen studies did not refer to participant attention levels.^5467 69 71 73^,^76 79 80 82^ In total, two studies formally assessed and stated participant attention levels. This was done using parent scales^66^ and a measure of cognitive functioning.^87^ The included studies did not assess or explore the child’s view subjectively (ie, whether the intervention was fun and engaging for them). However, one study did attempt to measure the child’s engagement through calculating the mean duration of intervention sessions and the percentage of ‘distraction time’ during an activity (when the child was not looking at the toys or the adults).^77^ One study also included the rating of ‘attention’ level, defining this as a level of attention to their peer, toy and general play area.^44 46 81^

Interventions delivered via parents, for both speech and language, optimised child engagement in naturalistic contexts. They did this through integrating technique use into everyday routines of interest to the individual child and family,^67^,^6971 72 84^ as well as into storybooks and play with toys selected by the child.^54 77^ One (language) study described a cultural adaptation of the intervention to make it more accessible, socially valid and engaging for children and parents outside of the dominant culture.^83^ There is an evidenced need for such ‘culturally responsive’ intervention studies, with traditional parent–child interaction-based approaches not appropriate for all families.^93^

The included papers did not aim to hypothesis-test any alternative explicitly labelled theories that related to both oral vocabulary and speech comprehensibility. However, this is not surprising considering that the primary focus of the papers was on measuring the effectiveness of intervention approaches rather than aligning with theory.

### Question 3: what evidence is there for the effect of interventions that incorporate these core characteristics of intervention techniques?

Due to inconsistency in effect measures and incompletely reported data, vote counting based on direction of effect has been used.^94^ Regular vote counting processes based on statistical significance are not advised due to serious limitations with underpowered studies.^94^ Therefore, a more useful vote counting approach, based on direction of effect, was conducted. This enabled the researchers to present a summary direction of effect for both outcomes (vocabulary and speech comprehensibility), thus providing an overview of effectiveness across the evidence base. The process for calculating direction of effect for each study and developing effect direction plot (table 3) was based on the instructions given in Boon and Thomson.^95^ For oral vocabulary interventions, this included stating the direction of effect for static vocabulary and fluid vocabulary when both outcomes were measured within the same study. A sign test was not conducted due to the small number of studies limiting its power.^96^ Thirteen studies, consisting of 17 interventions, met the inclusion criteria for synthesis of effectiveness.

**Table 3:** Effect direction plot for studies meeting the inclusion criteria for summary of effectiveness

Vocabulary interventions (ordered by control)				
Study (intervention group)	Comparator	Sample size of intervention group	Outcome domain 1: static language	Outcome domain 2: fluid language
(direct intervention)^66^	No treatment control	^8^	▲	N/A
(indirect intervention)^66^	No treatment control	^8^	▲	N/A
^68^	No treatment control	^12^	▲	▲
(monolingual intervention)^73^	No treatment control	^11^	▲	▲
(bilingual intervention)^73^	No treatment control	^9^	▲	▲
^78^	No treatment control (business as usual)	^40^	N/A	◄►
(parent intervention)^67^	Alternative parental intervention (non-language-based)	^9^	▲	▲
(individual intervention)^67^	Alternative parental intervention (non-language-based)	^8^	▲	▲
(bilingual group)^70^	Alternative non-vocabulary intervention group: mathematics	^52^	▲	N/A
(English-only group)^70^	Alternative non-vocabulary intervention group: mathematics	^45^	▲	N/A
^69^	‘Care as usual’ only	^30^	◄►	N/A
^71^	‘Business as usual’ group	^16^	▲	▲
^72^	No treatment control (but free to access treatment elsewhere)	^45^	▲	▲
^77^	No treatment control (but receiving other one-to-one SLT treatment elsewhere)	^20^	N/A	◄►

Speech interventions (ordered by control)				
Study	Comparator	Study design	Sample size of intervention group	Outcome domain 1: speech intelligibility
^75^	No treatment control	RCT	^65^	▲
^74^	No treatment control (both groups free to access additional SLT elsewhere)	RCT	^20^	▲
^76^	No treatment control (but free to receive SLT elsewhere)	RCT	^26^	▲

Effect direction: upward arrow ▲, positive health impact;, downward arrow ▼, negative health impact;, sideways arrow ◄►, no change/mixed effects/conflicting findings.

N/Anot availableRCTrandomised controlled trialSLTspeech and language therapy

Due to restrictions in study design, case series were excluded from vote counting. For the four included language intervention case series, three reported a positive effect on all children who participated^81 83 84^ and one reported an increase in vocabulary for four children but no intervention effects for two children.^85^ One SSD case series led to an improvement in speech intelligibility for three out of the five children.^54^

**Figure 4:**
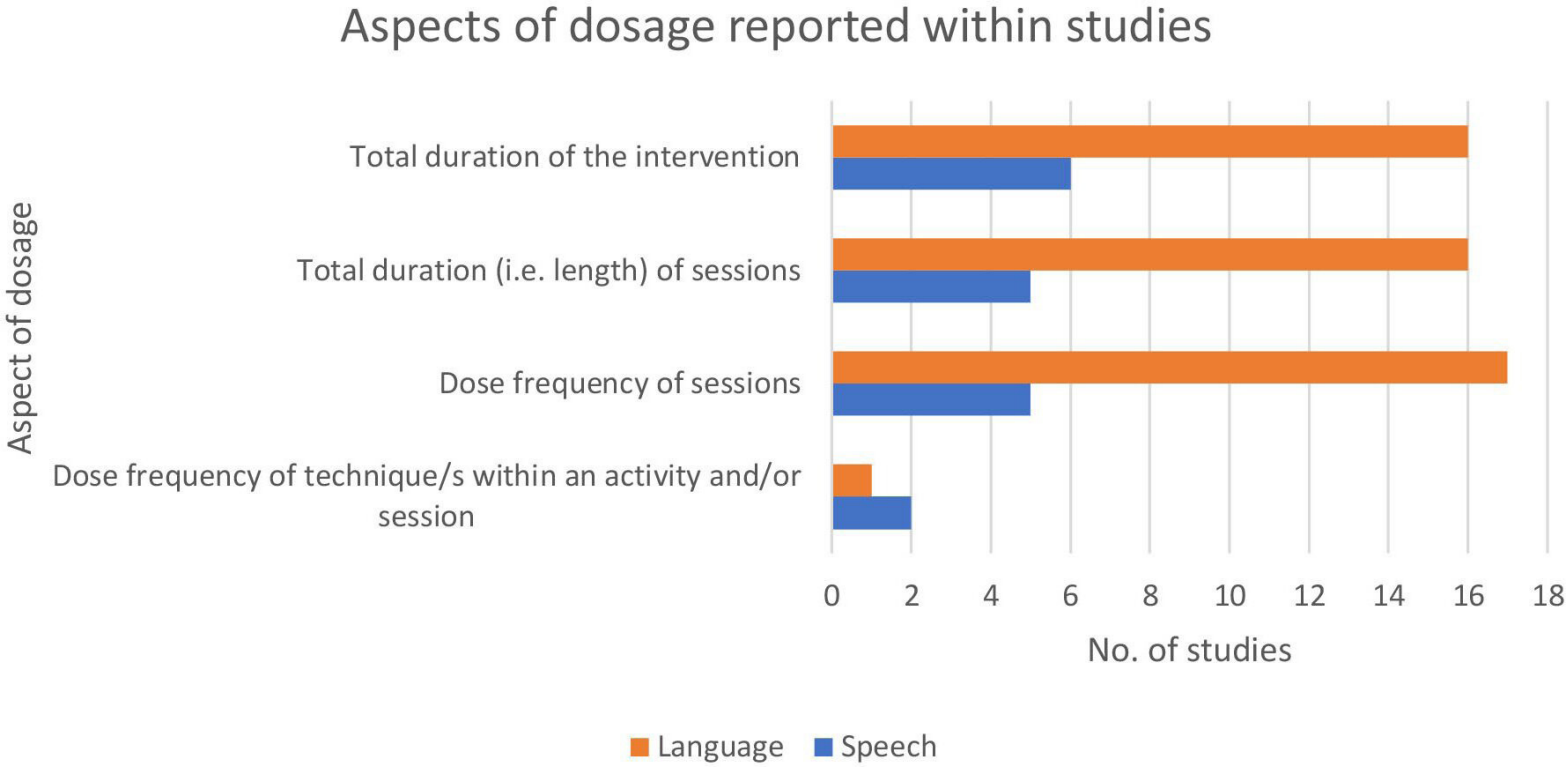
Aspects of dosage reported within studies.

As well as heterogeneity of study controls being a barrier to meaningful quantitative analysis, heterogeneity in the reporting of technique dosage within the individual interventions was also a factor in this. This heterogeneity can be viewed in figure 4.

## Discussion

This review has identified shared core characteristics of intervention techniques for developing speech (comprehensibility) and language (oral vocabulary), and how they relate to shared theories of relevance. Many of the techniques for speech and language are related to input (ie, the child hearing and processing words/sounds used by an adult) and were integrated into a variety of activities. Vote counting with direction of effect highlighted the positive direction of effect for most of the included studies. The principal findings in relation to clinical practice and theory development are elaborated on in the following sections.

### Similarities and differences

One key similarity between the language and speech interventions included the incorporation of techniques into a range of different activities, namely play, daily routines and storybooks. However, there was some variation in how play was conceptualised, with structured play being used more within the speech interventions and child-led play being used more in the language interventions. This coincides with findings from studies investigating clinical practice.^34^ A likely explanation for this is that many speech interventions rely on exposure to the specific sounds targeted; therefore, play might be structured in a way to incorporate item that provides optimal exposure.^54^ This has implications regarding the delivery of a combined speech and language intervention; should speech and language techniques be combined within the same play activity, consideration may have to be given regarding how to ensure this is predominantly child-led while maintaining maximal exposure to the child’s targeted sounds.

A second key similarity between the speech and language interventions was that, for those that involved training parents, the training used similar principles. This may be unsurprising given that the passing on of knowledge, practising and rehearsal opportunities are strategies that might be used in many adult learning scenarios, not just when supporting a parent to carry out speech and language techniques at home. Although there was adequate description of each method of training within the individual papers, the variable terms used to describe training methods were a barrier to establishing just how ‘like for like’ the training methods were. Ways to address this in the future might be to use universally applicable statements to describe ways that parents can be supported, for example by using predefined statements as provided in the behaviour change technique taxonomy, which is widely used within health research.^97^

### Implications for theory development

#### Lexical diversity and the lexical restructuring hypothesis

Vocabulary interventions were found to have a diverse range of outcomes and outcome measures, including ‘static’ measures (those that assessed specific vocabulary using a standard stimulus) and ‘fluid’ measures (those that assessed spontaneous language use in less structured activities). Within the lexical restructuring hypothesis, refinements to phonological representations take place under the weight of newly acquired vocabulary^39^; evidenced increases in either static or fluid vocabulary may therefore suggest a strengthening of phonological representations. It could be argued that fluid measures of vocabulary might be most indicative of the potential for this process having taken place, as it can encompass all items within the child’s lexical inventory rather than a select few. When considering applying this hypothesis to clinical practice, it would be premature to state that an increase in oral vocabulary categorically leads to clearer speech via the strengthening of phonological representations. Rather, we might cautiously hypothesise this, in view of the known link between phonological representations and speech production.^98^

#### Attention and engagement

A lack of information regarding the participants’ engagement and attention levels was noted across the language and speech studies. As highlighted within the multicomponent model of working memory,^44^ attention is inextricably linked to language learning and use. Children with co-occurring features of DLD/SSD are at increased risk for attention needs, including attention deficit hyperactivity disorder^99^; therefore it is unknown whether the included interventions have ‘implementation value’ to this often-seen group. Engagement is a different but closely related concept, as even for children with typically developing attention, motivation for them to engage within therapy activities is critical. This is why within both DLD and SSD interventions clinicians are required to tailor interventions according to the personality, engagement and attention levels of the children they are working with.^36 90^ Ease of transference between evidence and practice would be strengthened by including information on how interventions may be altered to optimise attention and engagement.

### Limitations of the review

#### Review focus and conduct

This review was theory-driven, and findings should be considered in view of both its strengths and limitations. A strong alignment with theoretical models does not categorically equate to effectiveness. Due to inconsistency of effect measures, incompletely reported data and variation in sample size, both significance and effect estimates could not be directly pooled and compared between studies. Although vote counting was carried out, this is a crude measure with limitations.^60^ Application of theory and the selection of appropriate measures to address this methodological gap are needed for theory testing and subsequent analysis of effectiveness. This might be achieved by, for example, using indirect measures of phonological representation^100^ when an increase in both lexical diversity and accuracy of speech production is observed.

A second limitation relates to the observed variability in how features of DLD and SSD are described within the included papers and the acceptance of this within the researchers’ review inclusion criteria. Some DLD studies communicated features clearly through specified criteria and used a range of assessments. In contrast, other studies indicated that their participants had features of DLD through one inclusion criteria alone, that their language needs existed with no known cause. Although full consensus between reviewers was achieved on abstract/full-text screening, this was a contributing factor to relatively low initial consensus. This variability in describing DLD/SSD also has implications for broader generalisation of knowledge gleaned, as to what degree the findings might apply to children with/without other characteristics (eg, receptive language difficulties) is uncertain. However, should inclusion criteria have been more stringent (eg, formal diagnosis of DLD using a variety of assessments), it is highly likely that potentially relevant studies would have been missed.

#### Limitations within included studies

There was variation in intervention reporting, as well as heterogeneity within comparator groups, which limited the extent to which interventions could be compared as ‘like for ‘like’. Comparator groups included no treatment controls and alternative intervention groups. Comparator type has been shown to be a significant influencing factor within oral vocabulary intervention research, with the observed effectiveness within interventions compared with a no treatment control being reduced when the intervention group is compared with other cognitive therapies instead.^101^ This reinforces the importance of reviewing each intervention study within the context of its comparator. This review of each intervention study individually is also necessitated by the paucity of dosage-related information across speech and language studies. Without quantifying intervention and technique dosage, comparing the effectiveness of different techniques across interventions remains challenging.^47^ These challenges are further exacerbated by variation in terminology for intervention techniques.

One final, but crucial, limitation of this review and knowledge base is that information deemed important to the expert steering group was missing and therefore could not be collected and analysed. This included whether the child had accessed interventions previously, whether they were in nursery and what their view of the intervention was. This under-reporting of information of significance to stakeholders has implications for the implementation of the interventions within real-world contexts.

### Recommendations

This review has provided important learning regarding intervention reporting and study design. We have generated recommendations in response to this:

- Lack of reported information (eg, dosage) and heterogeneity in study design, type of control and terminology continue to be a significant barrier to advancing knowledge in this area. Paediatric speech and language therapy interventions are complex by nature, with many interweaving components. To enable replication, aspects of the dose form framework^47^ should be used to supplement the reporting of intervention elements through the TIDieR checklist.^48^ A structured, robust approach to developing a core outcome set, as well as a standard diagnostic process and specification of interventions, is already underway in SSD,^102^ and similar work within DLD is emerging.^103^ By consistently implementing the findings from these projects, researchers will enhance the future implementation value of their work.
- Case series are highly beneficial to complex interventions, including those within the field of paediatric speech and language therapy; they enable the indepth exploration of processes and individual child responses. This methodology would be strengthened by using randomisation tests and between case effect sizes.^104^
- The child’s view of the intervention and levels of engagement may highlight why they do or do not respond to the intervention techniques used. Due to the significant communication barriers young children with DLD/SSD have, enabling them to communicate this has challenges. Although further work in this area is needed, researchers might consider measuring engagement through structured observations of the child’s behaviour.^105 106^
- ‘Buy in’ from families accessing interventions is crucial to success; there is no one-size-fits-all approach. In addition to the scientific effectiveness of interventions, there is an urgent need for future intervention research to be both purposeful and explicit about the social and cultural validity of the intervention being carried out. This is an important first step towards facilitating equity within paediatric speech and language therapy research.

These results should inform the development of new interventions for young children with co-occurring features of DLD and SSD, where oral vocabulary and speech comprehensibility are target areas. That no intervention studies were found to specifically address both of these outcomes further highlights the need for such interventions to be developed. Pertinent areas to explore further include the refinement of vocabulary as an outcome, including how it might be used to explore the lexical restructuring hypothesis. The similarities and differences identified between the DLD and SSD interventions should also be considered in relation to the feasibility of combining them into a single intervention.

In summary, this review has successfully identified the shared characteristics of intervention techniques for language (oral vocabulary) and speech (comprehensibility/intelligibility) and related them to relevant theory. The findings provide a foundation for a new intervention for vulnerable young children with co-occurring features of DLD and SSD. The review has also highlighted limitations within the current knowledge base, and future research may build on the recommendations given in this paper.

## supplementary material

10.1136/bmjopen-2023-081571 online supplemental file 1

10.1136/bmjopen-2023-081571 online supplemental file 2

10.1136/bmjopen-2023-081571 online supplemental file 3

10.1136/bmjopen-2023-081571 online supplemental file 4

10.1136/bmjopen-2023-081571 online supplemental file 5

10.1136/bmjopen-2023-081571 online supplemental file 6

## Data Availability

Data are available upon reasonable request.
