# Supplementary material for: Shared characteristics of intervention techniques for oral vocabulary and speech comprehensibility in preschool children with co-occurring features of developmental language disorder and speech sound disorder: a systematic review with narrative synthesis
Source: BMJ Open. 2024 Aug 28;14(8):e081571. doi: 10.1136/bmjopen-2023-081571 (PMC11367316; doi:10.1136/bmjopen-2023-081571)
Supplement: online supplemental file 2 [file bmjopen-14-8-s002.pdf]

<u>Extraction items</u>

Extraction items informed by the steering group are in **bold**

<u>Overarching study information:</u> Speech or language; title; author; year; country; language the intervention was in; study design; comparator of interest; no. participants; overarching study aim; outcome of relevance to this review.

<u>Participant information:</u> Age at baseline; sex; languages spoken; ethnicity; SES details; assessments used to identify their SSD/DLD features; phonological SSD subtype; how phonological SSD subtype indicated; **how bi/multi lingual children are assessed; previous speech and language therapy; family history; time in nursery; attention and listening levels.**

<u>Intervention information:</u> Setting; techniques; rationale; implicit or explicit; activities; dose frequency of the intervention; total duration of the intervention; dose frequency of techniques; total duration of the technique; other dosage information; technique deliverer; how deliverer is supported if non-professional; **flexibility of deliverer**; **how it was decided that the intervention was right for the child/family; was the intervention in the child's home language; how were parents supported; child's view of the intervention; if more than one deliverer, how were intervention tasks delegated.**

<u>Outcomes information:</u> Summary of exact outcome; measures used; timepoint/s; reported effect; direction of effect

<u>Metabias:</u> Registered protocol; if yes-was it registered prior to recruiting participants; if no-do the methods and results sections of the paper align.
