# Supplementary material for: Shared characteristics of intervention techniques for oral vocabulary and speech comprehensibility in preschool children with co-occurring features of developmental language disorder and speech sound disorder: a systematic review with narrative synthesis
Source: BMJ Open. 2024 Aug 28;14(8):e081571. doi: 10.1136/bmjopen-2023-081571 (PMC11367316; doi:10.1136/bmjopen-2023-081571)
Supplement: online supplemental file 3 [file bmjopen-14-8-s003.pdf]

Adapted GRIPP 2 Short Form

**Section and item**	**Where stated within the article**	**Further elaboration/additional information**
**Aim:** Aim of PPI in the study	To enhance social validity, make more relevant to the 'real world' (PPI section).	N/A
**Methods:** Clear description of the methods used for PPI in the study	Each steering group member had one to one meetings with the researcher as well as two whole group meetings which consisted of discussion and brainstorming (PPI section).	N/A
**Study results:** Outcomes- reported results of PPI in the study, including positive and negative outcomes	Creation of a shared extraction form, synthesis incorporates real world issues as well as those found in the literature (PPI and methodology sections).<br><br>Enabled commentary, reflection and action following the group discussions (PPI, results, and discussion sections).	The steering group raised many discussion points, due to time restraints these had to be prioritised.
**Discussion and conclusions:** Outcomes—Comment on the extent to which PPI influenced the study overall. Describe positive and negative effects	Supported with the extraction form and analysis. Pre-study PPI informed the outcomes (PPI and methodology sections).<br><br>The following was raised by the steering group- attention and listening, the child's perspective, social and cultural validity (PPI and discussion sections).	Negative effects- unable to extract information of importance to the steering group as often this was missing from the included papers.
**Reflections/critical perspective:** Comment critically on the study, reflecting on the things that went well and those that did not, so others can learn from this experience	N/A	Potential for more in depth involvement at 'leading' level e.g. being trained up to lead on quality appraisal.
