# Supplementary material for: Shared characteristics of intervention techniques for oral vocabulary and speech comprehensibility in preschool children with co-occurring features of developmental language disorder and speech sound disorder: a systematic review with narrative synthesis
Source: BMJ Open. 2024 Aug 28;14(8):e081571. doi: 10.1136/bmjopen-2023-081571 (PMC11367316; doi:10.1136/bmjopen-2023-081571)
Supplement: online supplemental file 4 [file bmjopen-14-8-s004.pdf]

Included studies

Table 1. Language studies, grouped by study design

Key: NDW = number of different words; MCDI = MacArthur Communicative Development Inventories (1); EOWPVT-3= Expressive One-Word Picture Vocabulary Test-3 (2); MLUw = Mean Length of Utterance in words; RWFT = Renfrew Word Finding Test (3); DLS-Derbyshire Language Scheme 1-word scores (4); Schlichting EL- Schlichting expressive language (5)

Study (country)	Study design	Quality Appraisal*	No. participants	Age range (at baseline)	Outcome of interest	Comparator	Techniques	Activities the techniques were used in	Deliverer	Technique location
**Gallagher, 2009 (UK)**	RCT	89%	Direct intervention-8; indirect intervention-8; no treatment control-8	3:7 to 4:2 years	Naming items in pictures (RWFT)	No treatment control	1)Modelling; 2) language recasting; 3) elicited imitation	Both intervention groups- storybooks- listening and retelling, action/movement, hide and seek (of toys representing targeted concepts). Direct intervention group only- 'did I say it	Direct intervention- SLT; indirect intervention- SLT and education staff	Direct intervention- child development centre; indirect intervention- nursery

								right?' games, free play		
**Gibbard, 1994 (UK)**	RCT	78%	Parental Intervention - 9; individual intervention - 8; alternative parental control-8	2:3 to 3:0 years	1)Naming items in pictures (DLS) 2) Parental subjective word list	Alternative parental intervention (non-language based)	Parental intervention 1) Modelling. Individual intervention group-not stated	Parental intervention-daily routines/situations, picture games, role play; individual intervention-not stated	Parental Intervention-parent; individual intervention-SLT	Parental intervention-trained at a health centre, technique use at home; individual intervention-health centre
**Girolametto et al. 1996 (Canada)**	RCT	78%	Intervention-12; no treatment control-13	1:1 to 2:11 years	1)NDW in play with parent; 2) parent report of vocabulary size (MCDI)	No treatment control	1) Modelling; 2)commenting;3) expansions; 4) repetition	Everyday routines; child led play	Parent	Home

**Kruythoff-Broekman et al. 2019 (Netherlands)**	RCT	78%	Intervention (also receiving care as usual)-30; care as usual only-30	2:0 to 2:5 years	Naming items in pictures (Schlichting EL)	Care as usual only group	1)Getting face to face; 2) wait and listen; 3) repeating words; 4) emphasising words; 5) expansion; 6) commenting; 7) choice questions; 8) reducing questions	Daily routines; child led play; songs	Parent	Parents trained at healthcare centre; techniques delivered at home
**Restrepo et al. 2013 (US)**	RCT	67%	Bilingual vocabulary group-52; English only vocabulary group-45	4:5 to 4:7 years (group means)	Naming items in pictures (specific intervention target words)	Four intervention groups compared to each other (bilingual vocabulary, English only vocabulary, bilingual mathematics,	Vocabulary intervention groups (English only and bilingual): 1) providing definitions; 2) scaffolding instruction; 3) modelling; 4) expanding (e.g. using novel words in new examples); 5) reviewing	Dialogic reading; story retelling; vocabulary book making; story acting; child led conversations	Trained professional (teacher)	Pre-schools/Head Start centres

						English only mathematics)				
**Roberts and Kaiser, 2012 (US)**	RCT	78%	Intervention-16; business as usual-18	2:7 years (mean for both groups)	NDW in play with parent	"Business as usual" language impaired group (92% did not receive language intervention)	1) taking turns; 2) waiting; 3) mirroring and mapping; 4) modelling specific language targets; 5) expanding verbal and non-verbal communication; 6)time delay strategies (assistance, choices, waiting with routine, waiting with cue, inadequate portions); 7) prompting strategies (open questions, choice questions, 'say' prompt)	Book reading; common household routines; snack; "toys"	Parent	Home and clinic

**Roberts and Kaiser, 2015 (US)**	RCT	78%	Intervention-45; no intervention control-52	2:6-2:7 (group means)	1)Parent report on language use *(MCDI))*; 2) NDW in play with parent; 3) naming one-word items in pictures (EOWPVT-3)	No intervention control (but free to receive SLT elsewhere)	1)Matched turns; 2) expansions; 3)time delays	Book reading; common household routines; snack; "toys"	Parent	Home and clinic
**Thordardottir et al. 2015 (Canada)**	RCT	100%	Monolingual intervention-11; bilingual intervention-9; no intervention	4:11 to 5:2 years (group means)	1)Use of targeted vocabulary when given probes; 2) MLUw from conversational sample	No intervention control group (delayed treatment)	1)Modelling; 2) repetition; 3) responsiveness to child's utterances; 4) positive reinforcement	Focused play; bingo; picture series depicting short stories	Monolingual intervention group-SLT only; bilingual intervention group-	Clinic

			control-9						SLT and parent	
**Lavelli et al. 2019 (Italy)**	Cohort	78%	Intervention-20; no intervention control-12	3:1 to 5:6 years	NDW in shared book reading activity with parent	No intervention control (but receiving other 1:1 SLT treatment elsewhere)	1)Modelling: contingent utterance, familiar topic utterance; 2) shared book handling; 3) captivating talking; 4) positive feedback; 5) bimodal utterance; 6) wh question + informative repair; 7)expansion	Shared book reading	Parent	Mixed-university, home, mixed university, home, child neuropsychiatry department

**Simon-Cereijido and Gutiérrez-Clellen, 2014 (US)**	Cohort	67%	Intervention-40; no intervention control-34	4:5 years (mean for both groups)	1)Mean Length of Utterance in words (MLUw-English and Spanish) in word picture book sharing with bilingual TA; 2) NDW (English and Spanish) in word picture book sharing with bilingual TA	No intervention control group (usual preschool curriculum)	1)Modelling; 2) mand-modelling; 3) balanced turn-taking; 4) wait-time; 5) expansions. New words introduced through: 6) definitions, 7) repetitions, 8) providing semantic associations.	Shared book reading in group time; small group activities	Teacher	School

**Craig-Unkefer and Kaiser 2003 (US)**	Case series (multiple baseline)	50%	6	3:1 to 3:11 years	NDW during play with peer	Multiple baseline	1)Modelling; 2) redirect with models; 3) redirect with direct instructions; 4) redirect with indirect instructions with hints; 5) reflective statements	Pretend play (adult scaffolded) including role play; manipulative play (e.g. construction); dramatic play (e.g. grocery shopping)	SLT	Head Start Centre
**Peredo et al. 2018 (US)**	Case series (multiple baseline)	50%	3	2:8 to 3:3 years	NDW in play/book sharing with parent	Multiple baseline	1)Matched turns; 2) modelling; 3) expansions; 4) time delays	Everyday routines; play; book sharing	Parent	Home
**Roberts et al. 2014 (US)**	Case series (multiple baseline)	50%	4	2:1 to 3:2 years	Use of target words in play with parent	Multiple baseline	1) Matched turns; 2) expansions; 3) time delays; 4) prompts (e.g. open questions, choice questions,"say" prompt)	Book reading; common household routines; snack; "toys"	Parent	Home and clinic

**Stanton - Chapman et al. 2008 (US)**	Case series	50%	6	3:9 to 4:5 years	Use of target words in play with peer	Multiple baseline	1)Labelling; 2) direct questions (e.g. what does this mean?' 3) using picture symbols; 4) modelling; 5) mands; 6) indirect instructions	Storybooks (designed so additional pages could be put in or taken out); play	Trained professional	Head Start Centre
**Hatcher and Page, 2020 (US)**	Case series (multiple baseline)	42%	4	2:10 to 3:8 years	NDW in play with parent	No multiple baseline for this outcome	1)Matched turns; 2) expansions; 3) time delays; 4) milieu prompting	Child led play	Parent	Home
**McGregor et al. 2020 (US)**	Case series	0%	7	4:10 to 6:6 years	Spontaneous target word production in journal sharing/ book reading and forced choice probes	No multiple baseline for this outcome	1)modelling; 2) elicitation; 3) child friendly definition; 4) contextualisation; 5) recontextualization	Within science instruction at boot camp; journal reflection; experiment/observation; text-based research	SLT	Science camp

**Deveney et al. 2014 (US)**	Case series (alternating treatments)	33%	3	2:1 to 2:9 years	Use of target words in response to probes during play	No multiple baseline for this outcome	1)Modelling; 2) language recasting; 3) expectant pause; 4) evoked production-cloze procedure	Play	The researcher	Home
**Weismer et al. 1993 (US)**	Case series (alternating treatments)	33%	3	2:1 to 2:2 years	NDW in response to probes during therapy activities	NDW in response to probes within the 'modelling' vs the 'modelling plus evoked production' contexts	1) Labelling; 2) modelling; 3) modelling + evoked production (modelling + evoked production context only)	Singing; turn taking games; what's in the box	SLT	Not stated

* Percentages taken from PEDRO-P (RCT/cohort) items 1-4 and 7-11; ROBiNT (case series) items 1-3 and 5-7

Table 2. Speech studies, grouped by study design

Key: ICS- intelligibility in context scale (6)

**Study (country)**	**Study design**	**Quality Appraisal***	**No. participants**	**Age range (at baseline)**	**Outcome of interest**	**Comparator**	**Techniques**	**Activities the techniques were used in**	**Deliverer**	**Technique location**
**McGill et al. 2020 (Australia)**	RCT	78%	Intervention-20; no treatment control-22	4:0 years (mean for both groups)	Parent report of intelligibility (ICS)	No treatment control (both groups allowed to access additional speech therapy elsewhere)	1)Modelling; 2)recasting; 3) expanding	Songs; rhymes; book sharing; play routines; daily routines	Parent	Home
**McLeod et al. 2017 (Australia)**	RCT	56%	Intervention-65; no treatment control-58	4:7 to 4:9 years (group means)	Parent report of intelligibility (ICS)	No treatment control	Not stated	Electronic games-phoneme factory sound sorter	Electronic device overseen by a professional	Early Childhood Education Centres

**Yoder et al. 2005 (US)**	RCT	78%	Intervention- 26; no intervention control- 26	3:7 to 3:8 (group means)	Speech intelligibility (from language sample analysis of play with a researcher)	No intervention control group (but free to receive SLT elsewhere)	1)Broad Target Recasts	Play	Trained professional (psychology graduate)	"Small therapy room"
**Sugden et al. 2020 (Australia)**	Case series (multiple baseline)	50%	5	3:3 to 5:11 years	Parent report of intelligibility (ICS)	Within subject (three timepoints)	1)Multiple oppositions- drill play (providing opportunities to hear and produce)	Naturalistic play activities with strategically selected toys; shared book reading	SLT and parent	University clinic; home
**Combiths et al. 2021 (US)**	Case series (multiple baseline)	33%	4	4:1 to 5:11 years	Parent report of intelligibility (ICS)	Pre/post (no multiple baseline for this outcome)	1)Eliciting production in imitation; 2)feedback on production; 3) multi-sensory cueing; 4)eliciting without a	Drill-play	SLT	Not stated

							model; recasting			
**Petinou and Theodorou m 2019 (Cyprus)**	Case study (multipl e baseline)	0%	1	4:6 years	Teacher report of intelligibilit y (ICS)	Pre/post only (no multiple baseline for this outcome)	Cycles approach-specific techniques not mentioned	Cycles approach-specific activities not mentioned	SLT	SLT's office premises

* Percentages taken from PEDRO-P (RCT/cohort) items 1-4 and 7-11; ROBiNT (case series) items 1-3 and 5-7

<u>References for supplementary materials 4</u>

1 MCDI: Fenson L, Dale P, Reznick JS, Thal D, Bates E, Hartung J, Pethick S, Reilly J. The MacArthur Communication Developmental Inventories. San Diego, CA: Singular. 1993.

2) EOWPVT-3/Expressive One-Word Picture Vocabulary Test: Brownwell R. Expressive One-Word Picture Vocabulary Test. 3rd ed. Novato, CA: Academic Therapy Publications; 2000

3) Renfrew Word finding test: Renfrew C. Word-finding vocabulary scale. Published by the author at North Place, Oxford, UK. 1988.

4) Derbyshire1 word scores: Masidlover M. The Derbyshire Language Scheme: Remedial teaching for language delayed children. Child: care, health and development. 1979 Jan.

5) Schlichting Expressive Language Test: SCHLICHTING, J. E. P. T. and LUTJE SPELBERG, H. C., 2010b, Schlicht-ing Test voor Taalproductie—II (Houten: Bohn Stafleu van Loghum).

6) ICS: McLeod S, Harrison LJ, McCormack J. 2012.The intelligibility in context scale: Validity and reliability of a subjective rating measure.
