# Supplementary material for: Shared characteristics of intervention techniques for oral vocabulary and speech comprehensibility in preschool children with co-occurring features of developmental language disorder and speech sound disorder: a systematic review with narrative synthesis
Source: BMJ Open. 2024 Aug 28;14(8):e081571. doi: 10.1136/bmjopen-2023-081571 (PMC11367316; doi:10.1136/bmjopen-2023-081571)
Supplement: online supplemental file 5 [file bmjopen-14-8-s005.pdf]

Validity ratings

PEDRO-P	McGill et al. 2020	McLeod et al. 2017	Yoder et al. 2005	Gallagher and Chiat, 2009	Gibbard, 1994	Girolametto et al. 1996	Kruythoff-Broekman et al. 2019	Lavelli et al. 2019	Restrepo et al. 2013	Roberts and Kaiser 2012	Roberts and Kaiser 2015	Simon-Cereijido, and Gutiérrez-Clellen, 2014	Thordardottir et al. 2015
Eligibility criteria were specified	Yes	No	Yes	Yes	Yes	Yes	Yes	Yes	Yes	Yes	Yes	Yes	Yes
Subjects were randomly allocated to interventions	Yes	No	Yes	Yes	Yes	Yes	Yes	No	Yes	Yes	Yes	No	Yes
Allocation was concealed	No	No	No	No	No	No	No	No	No	No	No	No	Yes
The intervention groups were similar at baseline regarding the most important prognostic factors	Yes	Yes	Yes	Yes	Yes	Yes	Yes	Yes	No	Yes	Yes	Yes	Yes
There was blinding of all subjects	No	No	No	No	No	No	No	No	No	No	No	No	No
There was blinding of all therapists who administered the therapy	No	No	No	No	No	No	No	No	No	No	No	No	No
There was blinding of all assessors who measured at least one key outcome	No	No	No	Yes	No	No	No	Yes	No	No	No	No	Yes
Measures of at least one key outcome were obtained for more than 85% of the subjects initially allocated to groups	Yes	Yes	Yes	Yes	Yes	Yes	Yes	Yes	Yes	Yes	Yes	Yes	Yes
All subjects for whom outcome measures were available received the treatment or control condition as allocated or, where this was not the case, data from at least one key outcome was analysed by "intention to treat"	Yes	Yes	Yes	Yes	Yes	Yes	Yes	Yes	Yes	Yes	Yes	Yes	Yes

The results of between-intervention group statistical comparisons are reported for at least one key outcome	Yes	Yes	Yes	Yes	Yes	Yes	Yes	Yes	Yes	Yes	Yes	Yes	Yes
The study provides both point measures and measures of variability for at least one key outcome	Yes	Yes	Yes	Yes	Yes	Yes	Yes	Yes	Yes	Yes	Yes	Yes	Yes
Total score	7	5	7	8	7	7	7	7	6	7	7	6	9

ROBINT	Combiths et al. 2021	Craig-Unkefer and Kaiser 2003	Petinou and Theodorou 2019	Sugden et al. 2020	Deveney et al. 2014	Hatcher and Page, 2020	McGregor et al. 2020	Peredo et al. 2018	Roberts et al. 2014	Stanton-Chapman et al. 2008	Weismer et al. 1993
Design with control	0	0	0	1	0	0	0	0	1	0	0
Randomisation	1	0	0	1	0	0	0	0	0	0	1
Sampling of behaviour	1	2	0	0	1	1	0	2	1	2	1
Blinding of people involved in the intervention	0	0	0	0	0	0	0	0	0	0	0
Blinding of assessors	0	0	0	0	0	0	0	0	0	0	0
Interrater agreement	2	2	0	2	2	2	0	2	2	2	1
Treatment adherence	0	2	0	2	1	2	0	2	2	2	1
Baseline characteristics	2	2	2	2	2	2	2	2	2	2	2
Setting	0	2	1	1	1	2	1	2	2	2	0
Dependent variable (target behaviour)	2	2	2	2	2	2	1	2	2	2	2
Independent variable (therapy/intervention)	1	2	0	1	2	1	2	2	2	2	1
Raw data record	0	1	0	1	0	0	1	0	0	0	0
Data analysis	2	0	0	2	2	0	0	0	0	0	1

Replication	2	2	0	2	1	2	0	2	2	2	1
Generalisation	2	1	0	1	2	0	0	2	2	1	0
Internal validity score	4	6	0	6	4	5	0	6	6	6	4
External validity score	11	12	3	12	12	9	7	12	12	11	7
Total score	15	18	5	18	16	14	7	18	18	17	11
