# Supplementary material for: Shared characteristics of intervention techniques for oral vocabulary and speech comprehensibility in preschool children with co-occurring features of developmental language disorder and speech sound disorder: a systematic review with narrative synthesis
Source: BMJ Open. 2024 Aug 28;14(8):e081571. doi: 10.1136/bmjopen-2023-081571 (PMC11367316; doi:10.1136/bmjopen-2023-081571)
Supplement: online supplemental file 6 [file bmjopen-14-8-s006.pdf]

Intervention techniques

Technique *(no. interventions)*	References
**Language**	
**Modelling *(15)***	Craig-Unkefer and Kaiser 2003; Deveney et al. 2014; Gallagher, 2009; Gibbard, 1994 (1 group); Girolametto et al. 1996; Lavelli et al. 2019 (*"modelling contingent utterance" + "familiar topic utterance")*; McGregor et al. 2020; Peredo et al. 2018; Restrepo et al. 2013 (both interventions); Roberts and Kaiser, 2012; Simon-Cereijido and Gutiérrez-Clellen, 2014; Stanton-Chapman et al. 2008; Weismer et al. 1993 (both intervention groups)
**Expansions *(9)***	Girolametto et al. 1996; Hatcher and Page, 2020; Kruythoff-Broekman et al. 2019; Lavelli et al. 2019; Peredo et al. 2018; Roberts and Kaiser, 2012; Roberts et al. 2014; Roberts and Kaiser, 2015; Simon-Cereijido and Gutiérrez-Clellen, 2014;
**Elicitation *(6)***	Gallagher, 2009 *("elicited imitation")*; McGregor et al. 2020; Roberts and Kaiser, 2012 and Roberts et al. 2014 *("open questions and "say" prompt")*; Stanton-Chapman et al. 2008 *("direct questions")*; Weismer et al. 1993 *("modelling with evoked production")* (1 intervention)
**Matched turns *(6)***	Hatcher and Page, 2020; Peredo et al. 2018; Roberts and Kaiser, 2012; Roberts et al. 2014; Roberts and Kaiser, 2015; Simon-Cereijido and Gutiérrez-Clellen, 2014 *("balanced turn taking")*;
**Time delays *(5)***	Hatcher and Page, 2020; Peredo et al. 2018; Roberts and Kaiser, 2012 and Roberts et al. 2014 (assistance, waiting with routine, waiting with cue, inadequate portions); Roberts and Kaiser, 2015
**Definitions *(4)***	McGregor et al. 2020 *("child friendly definitions")*; Simon-Cereijido and Gutiérrez-Clellen, 2014; Restrepo et al. 2013 (both intervention groups)
**Repetition *(4)***	Girolametto et al. 1996; Kruythoff-Broekman et al. 2019 *("repeating words");* Simon-Cereijido and Gutiérrez-Clellen, 2014; Thordardottir et al. 2015
**Choice questions *(3)***	Kruythoff-Broekman et al. 2019; Roberts and Kaiser, 2012; Roberts et al. 2014
**Contextualisation *(3)***	McGregor et al. 2020; Restrepo et al. *2013 ("using novel words in new examples")* (both intervention groups);
**Waiting *(4)***	Deveney et al. 2014 ("expectant pause"); Kruythoff-Broekman et al. 2019 *("wait and listen")*; Simon-Cereijido and Gutiérrez-Clellen, 2014 (*"wait time")*; Roberts and Kaiser, 2012
**Commenting *(2)***	Girolametto et al. 1996; Kruythoff-Broekman et al. 2019
**Labelling *(2)***	Weismer et al. 1993 (both intervention groups)
**Positive feedback *(2)***	Lavelli et al. 2019; Thordardottir et al. 2015 *("positive reinforcement")*

**Recasting (language) *(2)***	Deveney et al. 2014; Gallagher, 2009
**Biomodal utterance *(1)***	Lavelli et al. 2019
**Captivating talking *(1)***	Lavelli et al. 2019
**Cloze procedure *(1)***	Deveney et al. 2014
**Emphasising words *(1)***	Kruythoff-Broekman et al. 2019
**Getting face to face *(1)***	Kruythoff-Broekman et al. 2019
**Indirect instructions *(1)***	Stanton-Chapman et al. 2008
**Mand modelling *(1)***	Simon-Cereijido and Gutiérrez-Clellen, 2014
**Milieu prompting *(1)***	Hatcher and Page, 2020
**Mirroring and mapping *(1)***	Roberts and Kaiser, 2012
**Providing semantic associations *(1)***	Simon-Cereijido and Gutiérrez-Clellen, 2014
**Recontextualization *(1)***	McGregor et al. 2020
**Redirect with direct instructions *(1)***	Craig-Unkefer and Kaiser 2003
**Redirect with indirect instructions with hints *(1)***	Craig-Unkefer and Kaiser 2003
**Redirect with models *(1)***	Craig-Unkefer and Kaiser 2003
**Reducing questions *(1)***	Kruythoff-Broekman et al. 2019
**Reflective statements *(1)***	Craig-Unkefer and Kaiser 2003
**Responsiveness to child's utterances *(1)***	Thordardottir et al. 2015
**Reviewing *(1)***	Restrepo et al. 2013
**Scaffolding instruction *(1)***	Restrepo et al. 2013
**Shared book handling *(1)***	Lavelli et al. 2019
**Using picture symbols *(1)***	Stanton-Chapman et al. 2008
**'Wh' question + informative repair *(1)***	Lavelli et al. 2019

**Technique *(no. interventions***	**References**
**Speech**	

**Drill play *(2)***	Combiths et al. 2021 *("eliciting production in imitation")*; Sugden et al. 2020
**Recasting (speech) *(2)***	Combiths et al. 2021; McGill et al. 2020
**Broad target recasts *(1)***	Yoder et al. 2005
**Eliciting production without a model *(1)***	Combiths et al. 2021
Expansion (1)	*McGill et al. 2020 (unclear-possibly for language, not speech)*
**Feedback on production *(1)***	Combiths et al. 2021
Modelling (1)	*McGill et al. 2020 (unclear-possibly for language, not speech)*
**Multi-sensory cueing (visual, verbal and/or tactile) *(1)***	Combiths et al. 2021
