# Supplementary material for: Shared characteristics of intervention techniques for oral vocabulary and speech comprehensibility in preschool children with co-occurring features of developmental language disorder and speech sound disorder: a systematic review with narrative synthesis
Source: BMJ Open. 2024 Aug 28;14(8):e081571. doi: 10.1136/bmjopen-2023-081571 (PMC11367316; doi:10.1136/bmjopen-2023-081571)
Supplement: Supplementary file 7 [file bmjopen-2023-081571.reviewer_comments.pdf]

PEER REVIEW HISTORY

BMJ Open publishes all reviews undertaken for accepted manuscripts. Reviewers are asked to complete a checklist review form and are provided with free text boxes to elaborate on their assessment. These free text comments are reproduced below.

ARTICLE DETAILS

**TITLE (PROVISIONAL)**	Shared characteristics of intervention techniques for oral vocabulary a[illegible] co-occurring features of developmental language disorder and speech[illegible] synthesis
**AUTHORS**	Rodgers, Lucy; Botting, Nicola; Harding, Sam; Cartwright, Martin; Amer-El-khedoud, Meriem; Herman, Rosalind

VERSION 1 - REVIEW

**REVIEWER NAME**	Jucla , Mélanie
**REVIEWER AFFILIATION**	University of Toulouse
**REVIEWER CONFLICT OF INTEREST**	None
**DATE REVIEW RETURNED**	19-Jan-2024

**GENERAL COMMENTS**	This paper aims to review recent intervention studies (2012 to present) in young children with co-occurring DLD and SSD. The restriction of the outcomes of interest (vocabulary and speech intelligibility) is based on an interesting consensus approach. In fact, a group of parents and clinicians were asked to identify the most relevant measures of language/communication improvement. The rationale is clear and helps the reader to understand the importance of targeting comorbid DLD and SDD on the basis of a solid review of the literature. Based on a rigorous systematic review, the authors provide an overview of the methods used. They discuss the heterogeneity of measures/intervention techniques and contexts. The theoretical underpinnings of the studies are complemented by a meta-analytic analysis. Overall, this review is relevant to the field for both researchers and practitioners. Here are some comments that might help to clarify various points in the paper.<br><br>As mentioned above, the introduction is clear. We wondered why the author did not refer to the following review. Of course, its aim is different from that of the present study, but it provides recent findings that are relevant to the study.<br><br>Rinaldi, S., Caselli, M. C., Cofelice, V., D'Amico, S., De Cagno, A. G., Della Corte, G., ... & Zoccolotti, P. (2021). Efficacy of the treatment of developmental language disorder: A systematic review. Brain Sciences, 11(3), 407.<br><br>The authors often refer to the "technique of language modelling". As it is a core point of the paper, it would be interesting to give examples in the introduction section (page 6 of the manuscript) and in the results section (section Techniques and activities). Same thing for the "expansions" technique.

	Our main concern is with the third aim of the review. The authors want to link the results to theoretical accounts of language/speech processing. Although the introduction refers to the need for intervention programmes to be grounded in current theory, it is not clear whether the authors will analyse these links themselves or whether they will include studies that are theory-driven and then review what the studies say. We understood that this was not a research criterion when we read the methods section. And the results section confirms that the authors of the present study are carrying out what we have called above a meta-analytical analysis of how the results of the studies might be related to the theory. The authors should clarify and justify this choice in the introduction, the methods and the results section, as they do not review theory-based practice. Perhaps some of the studies base their intervention on a theory and the authors could provide information about this. For instance, at the end of the “Measuring vocabulary and the lexical restructuring hypothesis” section, the authors state “the studies did not aim to explore the lexical restructuring hypothesis”. Did they aim to test another hypothesis?<br><br>In the results section, the authors raise a point about 'child engagement and intervention context'. They consider the lack of assessment of attention, which is relevant given the high co-occurrence of ADHD and DLD. However, they also mention more motivational aspects. In our view, they could be more explicit about the potential contribution of both aspects and differentiate between them. This could be a point raised in the discussion section.<br><br>Finally, the discussion could have been more in-depth. Some of the points raised in the results could be included in the discussion. It would be useful to go into a little more detail on the question of the effects of context or the variability in the inclusion criteria in order to qualify the results and implications of the studies. Moreover, we understand none of the studies reported outcomes both in vocabulary and speech comprehensibility. Could the authors comment this point?<br><br>Typos : “consonsnants” (page 9 of the manuscript) – “by solely by” (section “Deliverer and setting”) – “hetergenity” (below table 3). There were formatting problems that may be independent of the authors. The figures and supplementary material at the end of the manuscript were not labelled, making it difficult to link text and figures. Pagination stopped after page 9 (manuscript).

**REVIEWER NAME**	Farquharson, Kelly
**REVIEWER AFFILIATION**	Florida State University
**REVIEWER CONFLICT OF INTEREST**	18-Apr-2024
**DATE REVIEW RETURNED**	I have no competing interests.

**GENERAL COMMENTS**	Thank you for the invitation to review the manuscript titled “Shared characteristics of intervention techniques for oral vocabulary and speech comprehensibility in pre-school children with co-occurring features of developmental language disorder and speech sound disorders: a systematic review with narrative synthesis”. This revew was completed alongside one of my doctoral students (Lydia Jiyoun Kim), to provide training experience. We reviewed the work

	independently and I have merged all relevent comments into this review. This is a critically important topic, as there are many preschool-aged children with co-occurring developmental language disorder (DLD) and speech sound disorder (SSD). Clinicians are often challenged to find interventions that allow for comprehensive and effective treatment for both disorders. This registered systematic review aims to identify intervention techniques related to the treatment of co-occurring idiopathic DLD and SSD, with a focus on the underlying theories that support the core intervention characteristics and their effectiveness. Available checklists and protocols (e.g., PRISMA, SWiM) are used throughout, and the authors use appropriate methods to address the research questions.<br>Introduction<br>The authors do a nice job of concisely describing and highlighting the importance of early treatment for both DLD and SSD. The authors review appropriate evidence to understand both disorders, and their common overlap. The outcome variables of interest are oral vocabulary for DLD and speech comprehensibility for SSD. More information is required to operationally define and justify these specific constructs.<br>While the section related to current interventions for co-occurring DLD and SSD adeptly outlines the current landscape of interventions and introduces valuable findings from 'Child Talk,' further refinement could enhance its coherence and applicability. Specifically, integrating a more detailed critique of existing interventions, smoother transitions between topics, and a clearer connection between theoretical insights and practical intervention strategies would significantly bolster the manuscript's contribution. Please clarify how the present review is different from the Child Talk systematic review. Is the present review intended to be an update of this work? If so, please specify that. This may also justify why the authors limited their search to articles dated 2012 and after. But it would be helpful to be clear about this.<br>The authors are interested in understanding how various intervention approaches may be related to three particular theories. The lexical restructuring hypothesis receives more attention in the literature review than the other two theories. In the discussion of the lexical restructuring hypothesis, a more specific conclusion that articulates the connection between vocabulary development and speech sound production is needed. While the hypothesis is mentioned as a basis for understanding the impact of expressive language growth on phonological skills, the current treatment stops short of fully elucidating how this theoretical framework informs practical intervention strategies for children with co-occurring DLD/SSD. To enhance reader comprehension and underscore the practical implications of this theory, I recommend the authors explicitly outline how improvements in vocabulary can lead to more accurate and refined speech sound production. Including a succinct, conclusive statement that directly links these concepts will not only solidify the theoretical discussion but also provide clear guidance for practitioners looking to apply these insights in clinical settings.<br>The psycholinguistic model is briefly explained, but the neural basis theory does not receive much attention. It would be helpful to add more information to the literature review to better contextualize all three theories and explicitly name them. That will provide more support leading into the research questions.<br>Page 7, line 57 – dose form framework is mentioned here, but requires much more information. Please explain the framework.<br>Method<br>The authors again mention Child Talk and that the criteria and

	search strategy for the present review are in line with it. It is important that the present review stand alone; readers may not be familiar with the Child Talk review and so the criteria should be explicitly listed here. Or a note that the specific criteria is detailed in the sections/ pages that that follow (e.g., the search strategy is on page 36 – direct the reader there). Description of study designs, population, intervention, comparator, and outcomes are clearly defined and appropriate to address the research questions.<br>Page 11 line 13, Covidence software is mentioned here for the first time. This should include a citation of the software and a brief description of it.<br>The data items, synthesis methods, and PPI statement are all clear and appropriate to address the research questions. In the risk of bias section, discussing the implications of the chosen threshold for inclusion in the analysis and the narrative synthesis approach in the context of this heterogeneity would reinforce the methodological rigor.<br>Results and Discussion<br>The tables and figures are clear and appropriate. It was not surprising that the oral vocabulary outcome measures were heterogenous. However, this is another reason that it will be important to better describe/ define oral vocabulary as the outcome measure of interest (as mentioned in the "introduction" section above). The transition between different types of measures and their implications for theory development seems abrupt. A clearer explanation of how each type of measure contributes to understanding oral vocabulary development in the context of the lexical restructuring hypothesis would be beneficial. Specifically, elaborating on how different vocabulary measures align with the lexical restructuring hypothesis.<br>For question 3 – the vote counting process is unclear. Please provide more detail regarding how this took place.<br>Table 3 – the header for the first column is confusing. (Intervention group) is listed in parentheses, but it appears that the data in that column include the study number in parentheses.<br>Figure 4 – aspects of dosage – these aspects need to be introduced and explained earlier in the manuscript. If these are examples "core characteristics", this can be set up in the introduction for clarity. At present, the introduction leads the reader to "who, where, and technique delivery" as the core characteristics. Since the dose form framework is not explained, this feels out of place. It is important however, so I recommend keeping it but just explaining it more clearly.<br>I recommend keeping the theories in the order that you've presented them in the introduction. Start with lexical restructuring. In general, the speech processing model is not well-explained in the introduction, so the discussion is difficult to follow as written.<br>The "child engagement/ attention" construct was surprising. While the section provides insightful observations on child engagement and intervention contexts, it would benefit significantly from a clearer articulation of the underlying theories that inform these aspects of intervention design. The implicit reliance on principles such as the importance of naturalistic learning environments and the child's active engagement could be more powerfully conveyed by directly connecting these intervention characteristics to established theoretical frameworks.<br><br>Minor line edits<br>Page 6, Line 33 – "became" should be "become"<br>Page 11 ,line 49 (and throughout) – consider referring to the original

	protocol as "the registered protocol for the present review". It includes more words, but the referent is more clear.<br>Page 12, line 10 – the word "firstly" is used here (change to "first"), but there is no second item listed. Please clarify.<br>In other systematic reviews, I have noted that authors use an * in the Reference list to indicate the citations that were associated with the review (in addition to having them in a table, like Table 1). I defer to the authors and editorial team on this, but it would have been helpful to me as a reader, particularly as the in-text citations are numbered.

VERSION 1 – AUTHOR RESPONSE

Reviewer 1: Dr. Mélanie Jucla, University of Toulouse

**Review comments**	**Addressing comments**
1.(The introduction) "did not include paper Rinaldi, S., Caselli, M. C., Cofelice, V., D'Amico, S., De Cagno, A. G., Della Corte, G., ... & Zoccolotti, P. (2021). Efficacy of the treatment of developmental language disorder: A systematic review. Brain Sciences, 11(3), 407".	Thank you for suggesting this interesting paper. We were aware of it, but noted that Rinaldi et al's review is much wider in scope (i.e. looking at all aspects of DLD, not just oral vocabulary) and with a wider age range (up to 8). Nevertheless, Rinaldi et al's findings are very interesting in the context of question 3 within our review (relating to effect of oral vocabulary interventions) and the impact of comparator. We have therefore incorporated this reference within the revised discussion section (1, page 24).
2."Techniques of modelling and expansion often referred to- would be helpful to have examples in the introduction (page 6) and results sections"	To make this clearer we have inserted some examples within both the introduction and results sections (2, pages 6 and 16).
3."Although the introduction refers to the need for intervention programmes to be grounded in current theory, it is not clear whether the authors will analyse these links themselves or whether they will include studies that are theory-driven and then review what the studies say".	The aim was to analyse these links ourselves as outlined in the introduction and methodology sections of our protocol.<br>https://bmjopen.bmj.com/content/13/6/e071262<br><br>However, we appreciate that this could be clearer within the current paper. We have therefore now stated that we will analyse the links ourselves within the aims section (3, page 8).
4."Perhaps some of the studies base their intervention on a theory and the authors could provide information about this. For instance, at the end of the "Measuring vocabulary and the lexical restructuring hypothesis" section, the authors state "the studies did not aim to explore the lexical restructuring hypothesis". Did they aim to test another hypothesis?"	Thank you for your excellent point. As stated in our response above, the aim was to analyse theoretical links ourselves, particularly as we were focused on theories linking SSD/DLD. However, we appreciate that the readership might be interested to know if the papers themselves mentioned any hypothesis testing of theories. We have therefore incorporated this into question 2 of our results section (4, page 18).

5."The authors raise a point about 'child engagement and intervention context'. They consider the lack of assessment of attention, which is relevant given the high co-occurrence of ADHD and DLD. However, they also mention more motivational aspects. In our view, they could be more explicit about the potential contribution of both aspects and differentiate between them. This could be a point raised in the discussion section".	We have incorporated our response into the discussion section, where we have stated that these aspects are both important, but might be conceptualised differently (5, page 23).
6."discussion could have been more in-depth. Some of the points raised in the results could be included in the discussion.	We have restructured the discussion, to incorporate additional discussion points arising from the results (6, page 22).
7."It would be useful to go into a little more detail on the question of the effects of context or the variability in the inclusion criteria in order to qualify the results and implications of the studies"(7)	We have also made some additional comments regarding the impact of variability in the inclusion criteria and effects of context on the implications of the results (7, pages 23-24)
8."none of the studies reported outcomes both in vocabulary and speech comprehensibility. Could the authors comment this point?"	Excellent point thank you. We have added this finding to our results and discussion sections (8, pages 14 and 25)

Typos/edits

"Typos : "consonsnants" (page 9 of the manuscript) – "by solely by" (section "Deliverer and setting") – "hetergenity" (below table 3)".	Corrected
"There were formatting problems that may be independent of the authors. The figures and supplementary material at the end of the manuscript were not labelled, making it difficult to link text and figures".	N/A journal formatting guidelines were followed for this
"Pagination stopped after page 9 (manuscript)".	Apologies, the page numbers were set to restart after page breaks. We have amended this.

Reviewer 2: Dr. Kelly Farquharson and Lydia Jiyoun Kim, Florida State University

**Reviewer comments**	**Addressing comments**
9."The authors do a nice job of concisely describing and highlighting the importance of early treatment for both DLD and SSD. The authors review appropriate evidence to understand both disorders, and their common overlap. The outcome variables of interest are oral vocabulary for DLD and speech comprehensibility for SSD. More information is required to operationally define and justify these specific constructs".	Thank you for this positive comment. We have understood it to mean that you would like to see how oral vocabulary and speech comprehensibility look operationally within clinical practice, as well as what improvements in them might look like. We have therefore added some information regarding this (9, pages 3 and 5)
10." It was not surprising that the oral vocabulary outcome measures were heterogenous. However, this is another reason that it will be important to better describe/ define oral vocabulary as the outcome measure of interest (as mentioned in the "introduction" section above)".	We have defined these constructs further within the outcomes section of the methodology, particularly in relation to 'fluid' and 'static' vocabulary, as these concepts are key to informing our later discussion regarding the lexical restructuring hypothesis (10, page 10)
11. "In general, the speech processing model is not well-explained in the introduction, so the discussion is difficult to follow as written".	We have amended our introduction to give more detail about the speech processing model (11, pages 5-6)
12. "Specifically, integrating a <u>more detailed critique of existing interventions,</u> smoother transitions between topic, and a clearer connection between theoretical insights and practical intervention strategies would significantly bolster the manuscript's contribution."	We have included commentary on relevant systematic review findings for speech/language, and provided further clarification regarding the impact of the child's age and developmental level on accessing interventions which already exist (12, pages 5-6)
13. "Specifically, integrating a more detailed critique of existing interventions, <u>smoother transitions between topic</u>, and a clearer connection between theoretical insights and practical intervention strategies would significantly bolster the manuscript's contribution."	Where possible, we have added in 'link' sentences to help bridge one paragraph from the next. We hope this makes it read more smoothly (13, pages 4-5)

14. "Specifically, integrating a more detailed critique of existing interventions, smoother transitions between topic, and a clearer connection between theoretical insights and <u>*practical intervention strategies*</u> *would significantly bolster the manuscript's contribution."*	We have added examples of intervention strategies and how they relate to the lexical restructuring hypothesis. We have also extended our explanation of the speech processing model and incorporated the multi component model of working memory into our neurodevelopmental theory section (14, pages 5-6).
15."Please clarify how the present review is different from the Child Talk systematic review".	We have added some additional information to address this point within the 'study overview and outcome' section (15, page 8). Specifically, how this review focuses on the outcomes of oral vocabulary and speech comprehensibility and relating them to underlying theory.
16. It would be helpful to add more information to the literature review to better contextualize all three theories and explicitly name them. That will provide more support leading into the research questions".	We have inserted a separate section for each theory and given examples of how they may be used within clinical practice (16, pages 5-6)
17. "Page 7, line 57 – dose form framework is mentioned here, but requires much more information. Please explain the framework.<br><br>*Figure 4 – aspects of dosage – these aspects need to be introduced and explained earlier in the manuscript. If these are examples of "core characteristics", this can be set up in the introduction for clarity. At present, the introduction leads the reader to "who, where, and technique delivery" as the core characteristics. Since the dose form framework is not explained, this feels out of place. It is important however, so I recommend keeping it but just explaining it more clearly".*	We have elaborated on what the dose form framework is within the 'study overview and outcomes' section and expanded on the different aspects of dosage within the introduction (17, pages 4,7,8).
18."It is important that the present review stand alone; readers may not be familiar with the Child Talk review and so the criteria should be explicitly listed here. Or a note that the specific criteria is detailed in the sections/ pages that that follow (e.g., the search strategy is on page 36 – direct the reader there)".	We have now added further clarification and signposting regarding this (18, page 9)
19."Page 11 line 13, Covidence software is mentioned here for the first time. This should include a citation of the software and a brief description of it".	A citation and brief description has been added (19, page 11)

20."In the risk of bias section, discussing the implications of the chosen threshold for inclusion in the analysis and the narrative synthesis approach in the context of this heterogeneity would reinforce the methodological rigor"	We have added further detail regarding heterogeneity in risk of bias levels (20, page 12)
21."For question 3 – the vote counting process is unclear. Please provide more detail regarding how this took place. Table 3 – the header for the first column is confusing. (Intervention group) is listed in parentheses, but it appears that the data in that column include the study number in parentheses".	We have elaborated on this and have revised the direction of effect table according to the instructions given in Boon and Thomson, 2021 (21, page 18). We hope that this is now much clearer.
22. "I recommend keeping the theories in the order that you've presented them in the introduction. Start with lexical restructuring".	We have re-formatted the discussion to give it a more logical sequence, whilst bearing in mind the word count. We have given the lexical restructuring hypothesis its own section as we have more commentary around this. (22, page 23 onwards)
23. "The transition between different types of measures and their implications for theory development seems abrupt. A clearer explanation of how each type of measure contributes to understanding oral vocabulary development in the context of the lexical restructuring hypothesis would be beneficial. Specifically, elaborating on how different vocabulary measures align with the lexical restructuring hypothesis.<br><br>*"In the discussion of the lexical restructuring hypothesis, a more specific conclusion that articulates the connection between vocabulary development and speech sound production is needed".*	We have now gone into more detail about different vocabulary measures and the lexical restructuring process. We have been cautious about making clinical recommendations based on our hypothesis, as the evidence base suggests that we need more evidence to support this first (i.e. from an intervention study which measures oral vocabulary, phonological representations, together with speech production) (23, pages 22-23)
24. "The "child engagement/ attention" construct was surprising. While the section provides insightful observations on child engagement and intervention contexts, it would benefit significantly from a clearer articulation of the underlying theories that inform these aspects of intervention design. The implicit reliance on principles such as the importance of naturalistic learning environments and the child's active engagement could be	Thank you for this important point. As a result of incorporating the multi component model of working memory into the introduction (24, page 6), we have been able to connect this to the results (24, page 18) and discuss our findings within the context of this (24, page 23).

more powerfully conveyed by directly connecting these intervention characteristics to established theoretical frameworks".	

Typos/edits

"*Minor line edits*<br>*Page 6, Line 33 – "became" should be "become"*<br><br>*Page 11 ,line 49 (and throughout) – consider referring to the original protocol as* ***"****the registered protocol for the present review****".*** *It includes more words, but the referent is more clear.*<br><br>*Page 12, line 10 – the word "firstly" is used here (change to "first"), but there is no second item listed. Please clarify".*	All items corrected<br><br>***"****the registered protocol for the present review****"*** – example on page 9
*"In other systematic reviews, I have noted that authors use an * in the Reference list to indicate the citations that were associated with the* review *(in addition to having them in a table, like Table 1). I defer to the authors and editorial team on this, but it would have been helpful to me as a reader, particularly as the in-text citations are numbered".*	This is a very good idea, we have added * to these references within the reference list. Regarding in text citations, as it is an online journal, they should appear (in full) when the reader clicks on the reference number.

VERSION 2 – REVIEW

**REVIEWER NAME**	Jucla , Mélanie
**REVIEWER AFFILIATION**	University of Toulouse
**REVIEWER CONFLICT OF INTEREST**	None
**DATE REVIEW RETURNED**	02-Jul-2024

**GENERAL COMMENTS**	The authors have done a commendable job of addressing the points I raised in my initial review. In my opinion, this paper should be published. There may be a minor error on page 18 (comment #4). The authors wrote "unsurprising." Did they intend to write "surprising" instead?

**REVIEWER NAME**	Farquharson, Kelly
**REVIEWER AFFILIATION**	Florida State University
**REVIEWER CONFLICT OF INTEREST**	I have no competing interests.
**DATE REVIEW RETURNED**	18-Jun-2024

**GENERAL COMMENTS**	The authors have addressed my comments and concerns from the initial round of edits. I have no further comments. Thank you for the opportunity to review this important work.

VERSION 2 – AUTHOR RESPONSE

Dr Farquharson and Dr Jucla- thank you once again for the time you have invested in reviewing this paper. Your thoughtful and detailed comments have been helpful in making it stronger.
